# Implementation of Group Physical Therapy for Knee Osteoarthritis: A Cluster Randomized Clinical Trial

**DOI:** 10.1001/jamanetworkopen.2025.35038

**Published:** 2025-10-02

**Authors:** Kelli D. Allen, Sara Webb, Cynthia J. Coffman, Livia Anderson, Graham Cummin, Connor Drake, Matthew Tucker, Amy Webster, Nina Sperber, Leah L. Zullig, Jaime M. Hughes, Lindsay A. Ballengee, Lauren M. Abbate, Helen Hoenig, Natalie Fullenkamp, Courtney H. Van Houtven, Susan N. Hastings

**Affiliations:** 1 Center of Innovation to Accelerate Discovery and Practice Transformation, Durham VA Health Care System, Durham, North Carolina; 2 Department of Medicine, University of North Carolina at Chapel Hill; 3 Thurston Arthritis Research Center, University of North Carolina at Chapel Hill; 4 Department of Biostatistics and Bioinformatics, Duke University Medical Center, Durham, North Carolina; 5 Department of Population Health Sciences, Duke University School of Medicine, Durham, North Carolina; 6 Department of Implementation Science, Wake Forest University School of Medicine, Winston-Salem, North Carolina; 7 Section on Gerontology and Geriatric Medicine, Division of Internal Medicine, Wake Forest School of Medicine, Winston-Salem, North Carolina; 8 VA Eastern Colorado Geriatric Research Educational and Clinical Center, Aurora; 9 University of Colorado School of Medicine, Aurora; 10 Center for the Study of Aging and Human Development, Duke University School of Medicine, Durham, North Carolina; 11 Department of Medicine, Duke University School of Medicine, Durham, North Carolina; 12 Physical Medicine and Rehabilitation Services, Durham VA Health Care System, Durham, North Carolina; 13 Geriatric Research, Education, and Clinical Center, Durham VA Health Care System, Durham, North Carolina

## Abstract

**Importance:**

Group-based physical therapy (PT) for knee osteoarthritis is an effective, efficient strategy for delivering care, but little is known about optimal strategies for implementing new clinical programs like group PT.

**Objective:**

To compare 2 implementation support approaches—foundational support and a combination of foundational support and more intensive enhanced support involving individual external facilitation—for delivering group PT.

**Design, Setting, and Participants:**

This cluster randomized clinical trial was conducted from January 31, 2022, to March 18, 2024, in outpatient PT departments at Department of Veterans Affairs (VA) Healthcare sites that agreed to deliver group PT to patients with symptomatic knee osteoarthritis. Sites were randomized to receive foundational or enhanced implementation support for delivering 6 sessions of group PT over 12 months that included exercise and educational content.

**Interventions:**

The foundational support approach included self-guided tools and monthly learning collaborative calls. The enhanced support approach added tailored support (eg, external facilitation) for sites that did not meet a priori benchmarks for adoption at 6 months or sustainment at 9 months.

**Main Outcomes and Measures:**

Site-level implementation outcomes were measured during months 7 to 12 and included penetration (primary outcome, assessed as the mean number of patients enrolled per month) and fidelity (mean number of classes attended per patient, out of a maximum of 6). Generalized linear models were used to examine differences in implementation outcomes between the enhanced and foundational support arms.

**Results:**

Nineteen sites (10 enhanced support, 9 foundational support) enrolling across 3 cohorts delivered group PT to 144 patients (68 enhanced support, 76 foundational support) during months 7 to 12. Patients were predominantly male (130 [90.3%]), with a mean (SD) age of 67 (9.2) years. Mean penetration estimates were 1.0 (95% CI, 0.2-1.7) patients enrolled per month for the enhanced support and 1.0 (95% CI, 0.1-1.9) for the foundational support arm, with an estimated mean difference between arms of −0.1 (95% CI, −1.1 to 1.0) patients enrolled (*P* = .92). Mean fidelity estimates were 5.0 (95% CI, 4.3-5.7) classes attended per patient in the enhanced support arm and 4.1 (95% CI, 3.2-4.9) in the foundational support arm, with an estimated mean difference between arms of 0.9 (95% CI, 0.0-1.9) classes per patient (*P* = .06).

**Conclusions and Relevance:**

In this cluster randomized clinical trial, an enhanced implementation support approach for delivering group PT did not outperform foundational support. Penetration was modest, illustrating the challenge of fostering referrals.

**Trial Registration:**

ClinicalTrials.gov Identifier: NCT05282927

## Key Points

**Question:**

Is an enhanced implementation support approach superior to less intensive foundational implementation support for promoting delivery of a group physical therapy program for knee osteoarthritis?

**Findings:**

In this cluster randomized clinical trial including 19 sites and 144 patients, sites receiving enhanced implementation support did not have significantly greater penetration (patients enrolled per month) or fidelity (number of classes per patient) compared with sites receiving foundational support. The rate of adoption was high for both arms, but penetration was relatively low.

**Meaning:**

In this study, enhanced implementation support did not outperform foundational support, suggesting different implementation approaches are likely needed to adequately support penetration.

## Teaser

This cluster randomized clinical trial compares the effectiveness of foundational vs enhanced implementation support for enrolling and retaining patients in a group physical therapy program for knee osteoarthritis.

## Introduction

Exercise-based physical therapy (PT), a key component of guideline-concordant care for knee osteoarthritis (OA), is important for mitigating functional decline.^1,2,3,4^ However, many individuals with knee OA do not receive PT.^5,6^ While reasons for underuse are multifactorial, a key challenge is the combination of high prevalence of knee OA and limited availability of PT services, particularly in lower-resource health care settings.^7^ Efficient models for delivering PT are needed to address this challenge. In a randomized clinical trial (RCT), group-based PT (*group PT* hereafter) yielded equivalent improvements in pain and functional outcomes compared with traditional individual PT among US veterans with knee OA.^8,9^ Because group PT requires fewer clinician hours per patient to deliver compared with individual PT, it can improve operational efficiency and enhance patient access.

Little is known about optimal strategies for supporting implementation of new clinical programs such as group PT in outpatient rehabilitation settings. When launching new programs, sites may experience challenges related to integration within existing clinic structures, learning how to deliver clinical services in a new format, and garnering appropriate patient referrals. Without appropriate support, sites may fail to successfully implement new evidence-based programs, hampering innovation and limiting use of interventions that could improve outcomes for patients and health systems. Building on prior work,^8,9,10^ we conducted a cluster randomized clinical trial comparing 2 different implementation support approaches to promote spread of group PT for veterans with knee OA. Specifically, we compared foundational implementation support with a combination of foundational support and enhanced support, a more intensive approach involving individual external facilitation, for sites that did not meet a priori benchmarks for group PT adoption or sustainment.^11^ Based on our study’s underlying conceptual model,^12^ implementation support intensification via practice facilitation is required to effectively facilitate self-organization and problem-solving to achieve improvements in implementation outcomes of interest.^13,14,15^ We applied a hybrid type 3 effectiveness-implementation framework, with a primary hypothesis that sites randomized to enhanced support would have superior implementation outcomes compared with those randomized to foundational support; we also compared patient-level outcomes between enhanced and foundational support sites.^12,16^ This study aimed to improve understanding of whether intensification of implementation support, tailored to site needs, offers an advantage over a standard, less intensive approach.

## Methods

This cluster randomized clinical trial (NCT05282927) was reviewed and approved by the VA Durham Health Care institutional review board, which waived informed consent because patient data collection was completed as part of clinical care. Detailed methods have been described previously.^12^ Quantitative results were reported using the Consolidated Standards of Reporting Trials (CONSORT) reporting guideline.^17^ The trial protocol appears in Supplement 1.

### Study Site Enrollment and Randomization

Department of Veterans Affairs (VA) Healthcare sites were recruited from January 1, 2022, through February 23, 2023, across 3 cohorts, 2 of which were clinical resource hubs, which provide gap coverage for multiple facilities.^18^ Site eligibility criteria were (1) availability of clinical personnel to deliver group PT, (2) active outpatient PT service, and (3) space to conduct in-person group sessions or capability to deliver group PT via telehealth. Sites committed to 12 months of participation, including providing staff to implement group PT and using a preprogrammed electronic health record (EHR) template. The final cohort concluded study activities in March 18, 2024.

Because site complexity and rurality may affect program implementation due to resource availability, randomization was stratified by these factors. Site complexity was determined by a VA facility-level measure, dichotomized as high (1a, 1b, or 1c) vs low (all other classifications).^19^ Rurality was based on the VA Office of Rural Health rurality calculator,^20^ dichotomized as facilities serving a population of 50% or more vs less than 50% rural or highly rural veterans. Stratified block randomization was used with sites randomized 1:1 to the foundational support or enhanced support arm. Sites were notified of their randomization assignment following the 6-month adoption benchmark assessment.

### Group PT Clinical Program

Group PT was based on prior work,^8,9,10^ best practices regarding PT and exercise for knee OA,^21^ and guidance from physical therapists and VA rehabilitation leaders. Group PT involved an initial PT evaluation followed by six 1-hour sessions including (1) collecting patient outcomes, (2) warm-up, (3) strengthening exercises (quadriceps, hips, hamstrings, step-ups, and calves) with 5 levels of difficulty, and (4) stretching and education. Beyond these essential elements, sites could customize the program, including the method of delivery (in-person or telehealth), personnel delivering the program (eg, physical therapist, PT assistant, or kinesiotherapist), number of patients per class (with a recommended maximum of 10), and enrollment of patients on a rolling basis vs in cohorts who started a series of classes together.

### Implementation Approaches

All sites received foundational support for 12 months, beginning at a specified start date. Foundational support was based on the Replicating Effectiveness Programs framework^22^ and conceptualized as a bundle of implementation strategies selected to address barriers to implementation success.^12,23,24^ Key foundational support resources included a detailed self-guided toolkit and monthly office hour calls; details are provided in eTable 1 in Supplement 2.

Sites randomized to enhanced support were evaluated 6 months after their start date for meeting the adoption benchmark of delivering at least 1 group PT class and enrolling at least 5 patients; those not achieving this benchmark then started receiving more intensive, tailored support. Sites were reassessed at 9 months to evaluate program sustainment, defined as enrolling at least 15 new patients during months 7 to 9. Sites that met the adoption benchmark but not the sustainment benchmark began receiving tailored support at that time. Therefore, sites could receive 3 or 6 months of enhanced support.

Enhanced support strategies featured implementation facilitation,^25^ defined as a multicomponent approach to improve the capacity of sites to address implementation gaps.^13^ Sites engaged in 1-to-1 calls with a trained implementation specialist (external facilitator) approximately every 3 to 4 weeks, with a maximum of 6 facilitation hours. The implementation specialist used collaborative strategies to help sites identify and address problems, with recommendations tailored to each site’s needs based on information gathered during each call. For example, if a site wished to increase patient referrals, the implementation specialist could gather information about past or ongoing efforts related to patient recruitment before working with the site to identify additional marketing and education activities.

### Measures

#### Implementation Outcomes

Site level implementation outcomes were collected from the VA EHR, with a primary time frame of months 7 to 12 (starting when enhanced support sites were eligible to receive more intensive support); outcomes were also assessed for the full 12-month implementation period. Penetration (the primary outcome), synonymous with the term *reach*, was defined as the mean number of patients enrolled per month. We also assessed the total number of patients enrolled. *Fidelity* was defined as the mean number of classes attended per patient with a maximum of 6. We also calculated the mean number of classes attended without capping the maximum at 6 (since sites could allow patients to attend more classes). For the fidelity outcome (as well as effectiveness outcomes), we included data from classes that occurred up to 15 months after the cohort start date to ensure that patients who enrolled later in the implementation period had an opportunity to attend at least 6 classes. The adoption outcome was defined as delivery of group PT and enrollment of at least 5 patients.

#### Effectiveness Outcomes

Patient outcomes, collected within group PT clinic notes, included a 30-second chair rise,^26^ patient-reported maximum pain during the chair rise (0 = “no pain,” 10 = “extreme pain”), Patient-Reported Outcomes Measurement Information System (PROMIS) Physical Function Short Form 4a score (range of 41.6-75.6, with higher scores indicating better function), PROMIS Pain Interference Short Form 4a score (range of 22.5-57.0, with higher scores indicating more pain interference),^27^ satisfaction with the group PT program (0 = “not at all satisfied,” 10 = “completely satisfied”), and ability to deal with daily problems with knee function and pain compared with before starting the group PT program, measured on a 5-point Likert scale (1 = “much worse,” 5 = “much better”).

Patient characteristics, including age, race, ethnicity, and birth sex, were extracted from the EHR. Race and ethnicity were included in the analysis to describe the study sample, along with other patient demographic and clinical characteristics. Race categories were American Indian or Alaska Native, Asian, Black, Native Hawaiian or Other Pacific Islander, White, and unknown. Ethnicity categories were Hispanic, Latino, or of Spanish origin; not Hispanic, Latino, or of Spanish origin; and unknown.

### Sample Size and Statistical Power

Sample size calculations were conducted for the primary outcome, penetration. Using a 2-sided *t* test with a type I error rate of 5%, a sample size of 16 sites yielded 80% and 90% power to detect effect size differences of 1.5 and 1.7 patients enrolled per month between arms, respectively. Based on the mean number of patients enrolled per month during a 12-month group PT implementation period at the Durham VA, with SDs from 1.5 to 2.5,^10^ these effect sizes corresponded to between-arm differences of 2.3 to 3.8 patients per month for 80% power and 2.6 to 4.3 patients per month for 90% power.

### Statistical Analyses

Generalized linear models were used to examine the effect of enhanced support vs foundational support on penetration and fidelity.^28^ Adoption is presented descriptively. For penetration and adoption, all sites were included in the intention-to-treat analyses; because fidelity was only relevant to patients who attended PT sessions, we excluded sites that did not launch a program. Models included indicators for study arm and stratification variables. Linear regression models were fit for penetration and fidelity outcomes. For total number of patients enrolled, a negative binomial regression model with a log link was fit.

For changes in patient outcomes (PROMIS pain interference and physical function scores, number of chair rises in 30 seconds, and pain during chair rises) from the first class to the last class, linear mixed models were fit for patients enrolled from 7 to 12 months. Models included indicators for study arm and class, interaction of arm by class, and the stratification variables. Random effects included site and site by class to account for clustering of patients within a site, and a compound symmetry covariance matrix was used to account for repeated measures within patients.^29^ Only outcomes from the last class were used for patient satisfaction and assessment of ability to deal with knee pain; linear regression models were fit for patient satisfaction and logistic regression models for ability to deal with knee pain. Models included arm and indicators for stratification variables with a random effect for site. Sensitivity analyses were conducted by fitting linear mixed models to patient outcomes of PROMIS pain interference and physical function scores, chair rises in 30 seconds, and pain during chair rises, including all classes that occurred up to 100 days after the first class. For these analyses, change in outcomes at 7 weeks (the mean number of weeks between the first and last class), as well as difference in change between arms and associated 95% CIs, were estimated from model parameters. Random effects included site and site by class, and random intercept and slope for time (from first class) were included to account for repeated measures. Models included indicators for arm, linear time, interaction variable of arm by linear time, and stratification variables.

Analyses were conducted using SAS, version 9.4 (SAS Institute Inc). Two-sided *P* < .05 was considered significant.

## Results

### Site and Patient Characteristics

Nineteen sites (10 receiving enhanced support and 9 receiving foundational support) were enrolled (surpassing our minimum goal of 16) across 3 cohorts with a range of 5 to 7 sites each. These sites delivered group PT to 189 patients during the full 12-month period and to 144 patients (68 enhanced support, 76 foundational support) during months 7 to 12, the primary analysis time frame. Of those 144 patients, 14 (9.7%) were female and 130 (90.3%) were male, with mean (SD) age of 67 (9.2) years. Two patients (1.4%) were American Indian or Alaska Native; 1 (0.7%), Asian; 23 (16.0%), Black; 4 (2.8%), Native Hawaiian or Other Pacific Islander; 97 (67.4%), White; and 17 (11.8%) had unknown race. Twenty-one (14.6%) were Hispanic, Latino, or of Spanish origin; 116 (80.6%), not Hispanic, Latino, or of Spanish origin; and 7 (4.9%) had unknown ethnicity (Table 1 and Table 2). The Figure shows the patient flow. The number of patients enrolled across sites in months 7 to 12 ranged from 0 to 20 (13.9%), with high variability across sites (coefficient of variation >80% in both arms). The mean (SD) number of days between patients’ first and last class was 50.7 (49.3).

**Table 1.** Site Characteristics, Total and by Randomization Arm

Characteristic	Sites, No. (%)		
	Enhanced support (n = 10)^a^	Foundational support (n = 9)	Total (n = 19)
Site’s implementation experience			
Quite a lot or a fair bit	7 (70.0)	8 (88.9)	15 (78.9)
Some	2 (20.0)	1 (11.1)	3 (15.8)
Very little or none	1 (10.0)	0	1 (5.3)
Facility complexity level^b^			
High	6 (60.0)	5 (55.6)	11 (57.9)
Medium or low	4 (40.0)	4 (44.4)	8 (42.1)
US geographic census region			
West	3 (30.0)	2 (22.2)	5 (26.3)
Midwest	2 (20.0)	0	2 (10.5)
Northeast	2 (20.0)	2 (22.2)	4 (21.1)
South	3 (30.0)	5 (55.6)	8 (42.1)
Rural facility^c^			
High	3 (30.0)	1 (11.1)	4 (21.1)
Low	7 (70.0)	8 (88.9)	15 (78.9)

^a^ One site withdrew after 6 months and did not receive enhanced support.

^b^ Defined using a Department of Veterans Affairs facility-level measure that reflects the complexity of services offered, with 5 classifications: 1a (most complex), 1b, 1c, 2, or 3 (least complex). We dichotomized complexity as high (1a, 1b, or 1c) vs low (all other classifications).

^c^ Determined using the Veterans Health Administration Office of Rural Health rurality calculator, which uses the closest facility or closest county or zip code to determine the percentage of rural veterans served. We dichotomized rurality as high (≥50%) and low (<50%).

**Table 2.** Patient Demographic Characteristics

Characteristic	Patients^a^		
	Enhanced support (n = 68)	Foundational support (n = 76)	Overall (n = 144)
Age, mean (SD), y	67 (8.6)	67 (9.8)	67 (9.2)
Sex assigned at birth			
Female	4 (5.9)	10 (13.2)	14 (9.7)
Male	64 (94.1)	66 (86.8)	130 (90.3)
Race			
American Indian or Alaska Native	1 (1.5)	1 (1.3)	2 (1.4)
Asian	1 (1.5)	0	1 (0.7)
Black	11 (16.2)	12 (15.8)	23 (16.0)
Native Hawaiian or Other Pacific Islander	3 (4.4)	1 (1.3)	4 (2.8)
White	45 (66.2)	52 (68.4)	97 (67.4)
Unknown	7 (10.3)	10 (13.2)	17 (11.8)
Ethnicity			
Hispanic, Latino, or of Spanish origin	10 (14.7)	11 (14.5)	21 (14.6)
Not Hispanic, Latino, or of Spanish origin	56 (82.4)	60 (78.9)	116 (80.6)
Unknown	2 (2.9)	5 (6.6)	7 (4.9)

^a^ Data are presented as number (percentage) of patients unless otherwise indicated. Primary outcome analyses included 144 patients who enrolled during months 7 to 12. A total of 189 patients enrolled in group physical therapy during the full 12-month study period.

**Figure.**
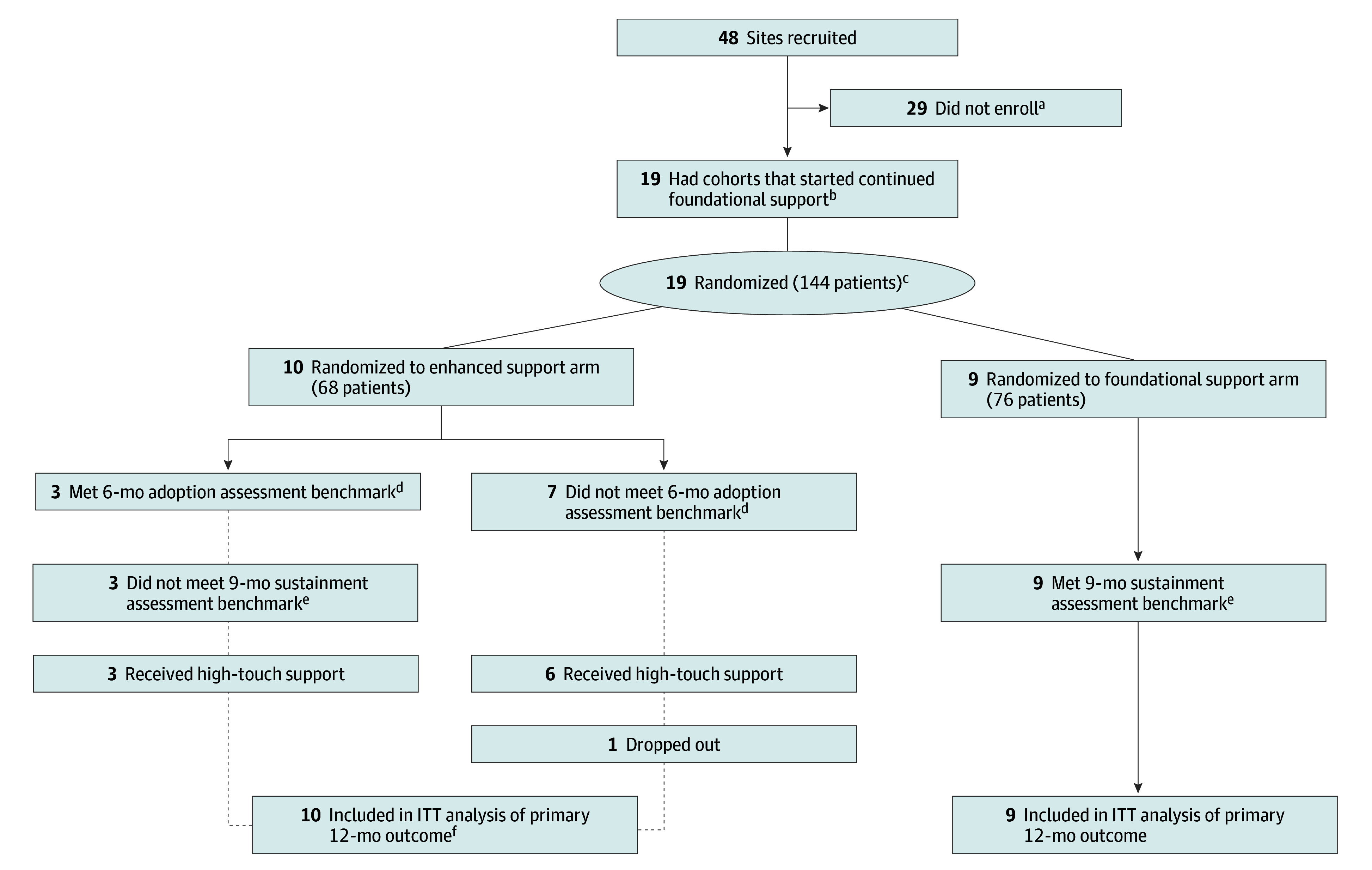
Group Physical Therapy (PT) Flow Diagram ^a^Reasons for not enrolling included loss to follow-up, change in clinic priorities, and staffing shortages. ^b^Regardless of randomization, all sites received low-touch supports for the study duration. ^c^A total of 189 patients enrolled during months 1 to 12, but analysis was conducted on the 144 who enrolled during months 7 to 12. ^d^Adoption assessment was defined as delivery of group PT as a clinical service (at least 1 class) and enrollment of at least 5 patients. ^e^Sustainment assessment was defined as enrolling 15 or more new patients between months 7 and 9. ^f^The site that withdrew from the study after the 6-month assessment was still included in the intention-to-treat (ITT) analysis.

### Group PT Delivery and Site Engagement With Implementation Strategies

Sixteen sites delivered a group PT program during the implementation period (9 of the enhanced support sites [56.2%] and 7 of the foundational support sites [43.8%]). Among these, 10 sites (62.5%) conducted in-person sessions (4 enhanced support sites [44.4%], 6 foundational support sites [85.7%]) and 6 (37.5%) conducted sessions via telehealth (5 enhanced support sites [55.6%], 1 foundational support site [14.3%]). Ten sites (62.5%) enrolled patients in cohorts (6 enhanced support sites [66.7%], 4 foundational support sites [57.1%]), and 6 (37.5%) offered rolling admission (3 enhanced support sites [33.3%], 3 foundational support sites [42.9%]).

The mean number of office hour calls attended per site was 6.6 (SD, 3.2; range, 1-11). Among the 10 enhanced support sites, 7 (70.0%) did not meet the 6-month adoption benchmark; 6 sites (60.0%) began receiving more intensive support, and 1 (10.0%) dropped out at that time. Of the 3 sites (30.0%) meeting the adoption benchmark, none met the 9-month sustainment benchmark. The mean numbers of facilitation calls received among sites beginning enhanced support at 6 months and 9 months were 5.5 (SD, 1.2; range, 4-7) and 3.3 (SD, 0.6; range, 3-4), respectively.

### Implementation Outcomes

#### Penetration

The mean penetration estimate for months 7 to 12 was 1.0 (95% CI, 0.2-1.7) patients enrolled per month for enhanced support and 1.0 (95% CI, 0.1-1.9) for foundational support, with an estimated mean difference between arms of −0.01 (95% CI, −1.1 to 1.0) patients (*P* = .92) (Table 3). The estimated mean total number of patients enrolled in months 7 to 12 was 5.2 (95% CI, 2.8-9.8) in enhanced support and 5.5 (95% CI, 2.7-11.3) in foundational support, with no difference between arms (rate ratio, 1.0; 95% CI, 0.4-2.2; *P* = .90). Similar results were found for months 1 to 12 (eTable 2 in Supplement 2).

**Table 3.** Estimated Means by Arm and Estimated Between-Arm Differences in Site-Level Implementation Outcomes From Months 7 to 12 Per Generalized Linear Models

Outcome	Estimated mean (95% CI), No.		Between-arm difference		
	Enhanced support (n = 10)	Foundational support (n = 9)	Estimated MD (95% CI)	RR (95% CI)	*P* value
**Penetration, patients enrolled, No.^a^**					
Monthly	1.0 (0.2-1.7)	1.0 (0.1-1.9)	−0.1 (−1.1 to 1.0)	NA	.92
Total	5.2 (2.8-9.8)	5.5 (2.7-11.3)	NA	1.0 (0.4-2.2)	.90
**Fidelity, classes attended, No.^b^**					
Out of 6 maximum	5.0 (4.3-5.7)	4.1 (3.2-4.9)	0.9 (0.0 to 1.9)	NA	.06
Total	6.2 (4.5-7.9)	4.3 (2.2-6.3)	2.0 (−0.4 to 4.3)	NA	.10

Abbreviations: MD, mean difference; NA, not applicable; PT, physical therapy; RR, rate ratio.

^a^ Primary outcome; analyses included 144 patients from 19 sites who enrolled during months 7 to 12.

^b^ Fidelity calculations only included sites that held a group PT class (9 in the enhanced support and 7 in the foundational support arm).

#### Fidelity and Adoption

The mean fidelity estimate for a maximum of 6 classes was 5.0 (95% CI, 4.3-5.7) classes per patient for enhanced support and 4.1 (95% CI, 3.2-4.9) for foundational support, with an estimated mean difference between arms of 0.9 (95% CI, 0.0-1.9) classes per patient (*P* = .06) (Table 3). When including classes above the maximum of 6 per patient, the estimated mean number of classes attended per patient was 6.2 (95% CI, 4.5-7.9) for enhanced support and 4.3 (95% CI, 2.2-6.3) for foundational support, with an estimated mean difference between arms of 2.0 (95% CI, −0.4 to 4.3) classes per patient (*P* = .10) (Table 3). Corresponding results for the 12-month implementation period are shown in eTable 2 in Supplement 2. During months 7 to 12, 6 enhanced support sites (60.0%) and 6 foundational support sites (66.7%) adopted group PT; 2 other enhanced support sites (20.0%) adopted group PT during months 1 to 6 (eFigure in Supplement 2).

### Effectiveness Outcomes

For PROMIS pain interference scores, there were improvements between the first and last class in both the enhanced support (−3.1 points; 95% CI, −5.7 to −0.5 points) and foundational support (−3.3 points; 95% CI, −6.0 to −0.6 points) arms (Table 4). For PROMIS physical function scores, there was no significant improvement between the first and last class in either the enhanced support (mean change, 1.5 points; 95% CI, −0.3 to 3.3 points) or foundational support (mean change, 1.5 points; 95% CI, −0.4 to 3.3 points) arm. For the number of chair rises completed in 30 seconds, there was an improvement in the enhanced support arm (mean change, 2.1 rises; 95% CI, 0.1-4.0 rises) but not in the foundational support arm (mean change, 2.5 rises; 95% CI, −0.6 to 4.4 rises). There were no significant changes in the mean maximum pain reported during the chair rise test for either arm. Mean ratings in satisfaction (scale of 0-10) at the last class were 8.8 (95% CI, 8.2-9.3) and 8.7 (95% CI, 8.3-9.3) in the enhanced and foundational support arms, respectively. The estimated rate of patients reporting that their ability to deal with knee pain problems was better at the last than the first class was 53.8% (95% CI, 32.4%-74.0%) in the enhanced support and 67.3% (95% CI, 44.2%-84.3%) in the foundational support arm (eTable 3 in Supplement 2). In the sensitivity analysis including all class data up to 100 days, inferential results were similar for all outcomes (eTable 4 in Supplement 2). There were no between-arm differences in any patient-level effectiveness outcomes.

**Table 4.** Estimated Means at First and Last Class and Changes From First to Last Class for Patient Outcomes During Months 7 to 12 and Estimated MDs in Change in Outcomes Between Arms From Linear Mixed Models^a^

Outcome	Enhanced support (n = 68)			Foundational support (n = 76)			Enhanced vs foundational support	
	Mean (95% CI)		Mean change (95% CI)	Mean (95% CI)		Mean change (95% CI)	Estimated MD in change (95% CI)	*P* value
	Baseline	Last class		Baseline	Last class			
PROMIS pain interference score^b^	61.7 (58.3-65.1)	58.5 (55.0-62.1)	−3.1 (−5.7 to −0.5)	60.4 (56.7-64.2)	57.1 (53.3-60.9)	−3.3 (−6.0 to −0.6)	0.2 (−3.5 to 3.9)	.90
PROMIS physical function score^c^	37.9 (35.5-40.2)	39.4 (37.0-41.8)	1.5 (−0.3 to 3.3)	38.8 (36.4-41.3)	40.3 (37.8-42.8)	1.5 (−0.4 to 3.3)	0.0 (−2.5 to 2.6)	.98
Chair rises in 30 s, No.^d^	10.1 (7.9-12.2)	12.2 (9.9-14.4)	2.1 (0.1 to 4.0)	9.5 (7.3-11.7)	12.0 (9.8-14.2)	2.5 (−0.6 to 4.4)	−0.5 (−3.1 to 2.1)	.70
Maximum pain during chair rise^e^	4.3 (3.0-5.6)	4.0 (2.6-5.4)	−0.3 (−1.5 to 1.0)	3.8 (2.3-5.2)	3.9 (2.4-5.3)	0.1 (−1.2 to 1.4)	−0.4 (−2.2 to 1.4)	.66

Abbreviations: MD, mean difference; PROMIS, Patient-Reported Outcomes Measurement Information System.

^a^ The study included 144 patients across 16 sites (9 enhanced support, 7 foundational support); 3 sites (1 enhanced support, 2 foundational support) did not deliver group physical therapy to any patients.

^b^ Score range, 22.5-57.0, with higher scores indicating more pain interference. Eleven patients were missing at the first class (4 enhanced support, 7 foundational support) and 31 at the last class (15 enhanced support, 16 foundational support).

^c^ Score range, 41.6-75.6, with higher scores indicating better physical function. Thirteen patients were missing at the first class (7 enhanced support, 6 foundational support) and 31 at the last class (14 enhanced support, 17 foundational support).

^d^ Eleven patients were missing at the first class (6 enhanced support, 5 foundational support) and 36 at the last class (15 enhanced support, 21 foundational support).

^e^ Measured on a scale of 0 (no pain) to 10 (maximum pain). Eleven patients were missing at the first class (3 enhanced support, 8 foundational support) and 30 at the last class (14 enhanced support, 16 foundational support).

## Discussion

This trial is one of the first we are aware of to directly compare different implementation approaches to support delivery of a new clinical program in outpatient PT. Contrary to our hypothesis, we found no between-group differences in implementation outcomes between foundational and enhanced support sites. There were also no differences in effectiveness outcomes between study arms, but there were improvements among patients overall, and patient satisfaction was high. Participation in office hour calls was reasonable, but there was considerable variability in engagement across sites. In the enhanced support arm, site engagement with the external facilitation calls was high, averaging about 1 call per month.

One potential reason for the lack of difference in implementation outcomes between study arms may have been the level of support provided by our foundational approach. All sites were provided with a comprehensive toolkit, monthly office hours with other sites, and an implementation specialist. Although the foundational support arm did not include individualized external facilitation, sites could share their specific challenges during office hours and receive input. This type of foundational support may be adequate to support adoption, which was fairly high for both study arms. Our data suggest that simply providing more intensive individualized support (particularly 1-to-1 calls with an external facilitator) for sites not meeting benchmarks may not improve implementation outcomes.

Penetration was low for both study arms. A key advantage of group PT is efficiency of delivering therapy in a group format, and a consistent stream of patients is important to realize this benefit. Study results illustrate the challenge of developing a pathway for regular patient referrals, which can require actively marketing a new program and ensuring that patient eligibility criteria and program benefits are effectively communicated.^30^ The current study’s results also suggest our enhanced approach did not adequately address penetration; further efforts are needed to develop strategies that specifically support sites in achieving optimal patient reach.

Although the rate of adoption was high across study arms, there was considerable variability in the time to launch group PT, from 3 to 10 months (eFigure in Supplement 2). Common factors delaying launch included installing the EHR note, integrating the program within the clinic schedule, and enrolling enough patients. Fidelity was high, with mean attendance of about 5 out of 6 sessions. This supports the feasibility of providing patients with an adequate dose of PT though a group-based program. Although there were small between-group differences in fidelity, favoring enhanced support, the lack of between-group differences in patient-level effectiveness outcomes suggests these differences were not clinically meaningful.

There were clinically meaningful improvements in patient-level effectiveness outcomes in both arms in PROMIS pain interference scores and in chair rises in the enhanced support arm.^27,31^ Our findings show that the positive impacts observed in an RCT^8^ and in single-site implementation of group PT^10^ persisted when the program was rolled out in a diverse group of sites with more limited governance over delivery processes and patient selection.

### Limitations

This study has limitations. It was conducted within the VA health care system, and further research is needed in other contexts. The primary period of observation was only 12 months, and longer-term outcomes are needed to fully understand the impacts of different implementation approaches for group PT delivery. It was not possible to determine the number of patients at each site who were appropriate for PT for knee OA, and data were not available regarding patients who declined participation in group PT when it was offered; this limits our understanding of penetration. In addition, sites’ use of each component of the implementation packages was not documented.

## Conclusions

In this cluster randomized clinical trial, enhanced implementation support did not outperform foundational support for delivery of group PT for knee OA. Group PT adoption was high, although penetration was low overall, illustrating the challenge of fostering referrals at some sites.

## Notes

Supplement 1.
Trial Protocol

Supplement 2. **eTable 1.** Description of Foundational and Enhanced Implementation Support Packages **eTable 2.** Estimated Means by Arm and Estimated Differences in Site-Level Implementation Outcomes During Months 1 to 12 Between Arms With Associated 95% CIs From Generalized Linear Models **eFigure.** Adoption Status and First Group PT Class Delivered by Month **eTable 3.** Estimated Mean at Last Class (n = 130 With Last Class) by Arm and Estimated Difference in Outcomes Between Enhanced Support vs Foundation Support With Associated 95% CIs From Generalized Linear Models **eTable 4.** Estimated Mean at Baseline and 7 Weeks for Patient Outcomes and Estimated Mean Difference in Change in Outcomes Between Arms With Associated 95% CIs When Including All Class Data up to 100 Days After First Class From Linear Mixed Models

Supplement 3.
Data Sharing Statement
