# Supplementary material for: Implementation of Group Physical Therapy for Knee Osteoarthritis: A Cluster Randomized Clinical Trial
Source: JAMA Netw Open. 2025 Oct 2;8(10):e2535038. doi: 10.1001/jamanetworkopen.2025.35038 (PMC12492051; doi:10.1001/jamanetworkopen.2025.35038)
Supplement: Supplement 1. — Trial Protocol [file jamanetwopen-e2535038-s001.pdf]

DVAHCS REQUIRED ELEMENTS AND PROTOCOL TEMPLATE

PROTOCOL TITLE: *Optimizing Function and Independence QUERI (Function QUERI 2.0) Function QUERI 2.0: Optimizing Function and Independence*

PRINCIPAL INVESTIGATOR: *S. Nicole Hastings, MD, MHS (clinician)*

VERSION NUMBER AND DATE: *V15 5/16/2024*

SPONSOR/FUNDING SOURCE*:* *VA QUERI*

Purpose

The overall impact goal of the Optimizing Function and Independence QUERI program ("Function QUERI") is to improve VA care by implementing and evaluating evidence-based clinical programs (EBPs) to maximize function and independence in vulnerable Veterans. Function QUERI's work directly addresses VA's 2018–2024 strategic goals and priorities related to Modernization: 1) Improve timeliness of services: ***Group PT improves access to care***; 2) Commit to zero harm: ***STRIDE reduces preventable harm of hospital-associated disability***; and 3) Implement the VA MISSION Act: ***Caregivers FIRST extends caregiver support services***, a direct mandate of the MISSION Act.

We will accomplish Function QUERI's overall goals through the following specific aims:

1) Implement 3 EBPs (**STRIDE, Caregivers FIRST**, and **Group Physical Therapy [PT] for Knee Osteoarthritis**) that have been recognized by VA leaders as high priority programs to fill quality gaps for Veterans at risk for functional decline or disability and their family caregivers;
2) Assess EBP impact on quality metrics and conduct business case analysis to inform sustainment;
3) Advance application of implementation strategies by testing implementation intensification approaches across EBPs;
4) Partner with VA leaders to conduct rapid high-quality implementation and evaluation projects to inform decisions on policies and practices to support optimal Veteran function and independence; and
5) Train the next generation of implementation science investigators who will become leaders in accelerating the adoption of evidence-based practices in VA.

Working in concert with our primary partners in VA's Office of Geriatrics and Extended Care, Diffusion of Excellence, Office of Caregiver Support, Physical Medicine and Rehabilitation, VISNs 6, 12 and 23, Veterans and family caregivers, Function QUERI will achieve an immediate impact on care within VA by providing access to EBPs for a large group of vulnerable Veterans at risk for functional decline. Long-term impacts will be to enhance VA's capacity to rapidly adopt evidence-based practices through advancing understanding of how to scale proven implementation strategies for national dissemination efforts and building VA's implementation capacity and workforce.

Optimizing Function and Independence Quality Enhancement Research Initiative

Durham VA IRB #2334

Protocol Modifications

**Summary of changes**
Initial Submission – approved on 12/23/2020 by the Durham VA IRB
The purpose of this submission (AMD 1) is to submit the draft staff survey for subproject Caregivers FIRST, part of Aim 1 working with the national Caregiver Support Program to “prepare and field a brief national survey to assess site organizational readiness specific to Caregivers FIRST rollout”.
1. IRB study title. We request to officially change our IRB name for 02334 from “Optimizing Function and Independence QUERI” to “Function QUERI 2.0: Optimizing Function and Independence” in order to not duplicate the name from our previous study (02040).<br>2. New document submission (STRIDE subproject, Aim 1): Data collection instruments including pre- and post-implementation surveys and qualitative interviews for staff, as well as an adaptations survey (pre-launch, 6 and 10 months) for site Point of Contact.<br>3. Updated language in protocol to increase staff survey participation, the study team may offer incentives (entry into a raffle for small prizes).
1. Updated language in protocol:<br>a. Page 7: updated implementation evaluation data sources to include in addition to field and call notes, polls from meetings we do virtually with sites (e.g., Poll Everywhere, Webex)<br>b. Page 13: For STRIDE subproject, updated study design and methods section, including:<br>i. updates to the recruitment plan for enrolling sites – now in in blocks every three months or when a threshold of sites is met (not exceeding 8), still with the overall plan to just enroll 32 total sites;<br>ii. adjustments to how we are randomizing sites (from covariate constrained randomization to stratified randomization)<br>iii. updates the site-level covariates we are collecting for randomization<br><br>2. Protocol deviation:<br>a. After consulting with ISO Scott Gardiner about using Poll Everywhere during calls with sites to gather information about implementation, the team had already conducted one field call, so we are submitting a protocol deviation. Scott Gardiner had recommended the study team amend the protocol to include polls and the software that is being used.<br>b. Since this study is exempt and does not require continuing review, we are adding this to our amendment submission.<br><br>3. Revisions to STRIDE subproject data collection instruments:<br>a. STRIDE Pre-implementation survey: minor instructions/wording changes from internal and field testing.<br>b. STRIDE Post-implementation survey: Page 8, we accidentally left off a measure question for Organizational Resilience. Also some minor instructions/wording changes from internal and field testing.<br>c. STRIDE Adaptations form: We are providing clarification that the adaptations data collection form submitted in this Amendment is for use in tracking program adaptations for process improvement purposes. We have updated instructions and protocol wording accordingly.<br>d. STRIDE pre-implementation qualitative interview guide: minor instructions/wording changes.

1. NEW Caregivers FIRST subproject data collection instruments:
a. Caregivers FIRST Field Readiness Survey: This instrument is very similar to the Field Readiness Survey submitted as part of Amendment 1. However, this survey will be fielded again to Caregiver Support Program managers this Fall, and we have made some minor wording changes (since we are assessing a new fiscal year).

Continuing Review

1. Revised language to the Enhanced Rep (enREP) activities (pg.6). This higher intensity support for project implementation will include 3 to 4 tailored modules including a barrier/needs assessment and facilitation, a process of interactive problem solving and support.
2. Updated study design and methods for Group PT (pg. 11), including site exclusion criteria, criteria for determining site rurality, and plans for randomization of sites to study arm.
3. Updated implementation activities for Group PT (pg.12), including updated adoption benchmarks.
4. Updated patient sample for Group PT (pg. 12). Patients do not need to have diagnosis by imaging for knee osteoarthritis or be >50 years of age to join Group PT classes at participating VAMCs.
5. Updated outcomes for Group PT (pg.12), including change in patient-level metrics, use of VA approved Qualtrics survey platform, and plans to collect data from all Group PT visits (instead of first and last visits).

1. Protocol updates:
• Pg. 6: Refined language to the Enhanced Rep (enREP) activities). Further detailing call procedures and how facilitators will help sites address barriers.
• Pg. 10: Added sustainment goals to how Group PT and STRIDE for sites randomized to receive higher implementation support.
• Pgs. 11-13: Group PT subproject: Further specifying procedures, including primary outcomes, site randomization variables, and implementation activities (including how team will use a data dashboard from CDW data to provide non-PHI/PII site-specific summary-level reports of Group PT activities to enrolled sites).
• Pg 14. STRIDE subproject
o Added thank-you gift bags for enrolled sites after study completion (sites will be unaware they will receive until end of study).
• Pgs. 16-17: Caregivers FIRST subproject
o Inclusion change for eligible sites - added low enrollment to adoption definition. Revised implementation support activities to align with our overall enREP changes.
• Pg. 26: Updated stratification variable analysis for Group PT.

3. Amendments to Group PT subproject data collection instruments:
• Group PT Pre-Implementation Staff Interview Guide
  o Updated title for Implementation Specialist.
• Group PT Post-Implementation Staff Interview Guide:
  o Shortened survey, updated title for Implementation Specialist, and inclusion of telehealth delivery questions (#7-9)
• Group PT Post-Implementation Staff Survey:
  o Slight modification to survey introduction.
• Group PT Baseline Adaptations form:
  o Change to Q1 answer choices and wording of several other questions.
• Group PT 6 Month Adaptations form:
  o Change to introductory language and other changes to align with revision in baseline adaptations form.
• Group PT 12 Month Adaptations form:

o Change to introductory language and other changes to align with revision in baseline adaptations form.<br><br>4. Amendment to STRIDE subproject data collection instruments:<br>• STRIDE Post-Implementation Staff Interview Guide: Minor change to intro and coordinator contact, moved and modified questions on stakeholders, modified question on site implementation, updated implementation strategy language, and slight modification to sustainability question wording.<br><br>5. NEW Caregivers FIRST subproject data collection instruments:<br>• Caregivers FIRST Pre-Implementation Staff Survey: Quantitative measures to assess teams' ability to successfully implement an evidence-based program (e.g., team size and composition, organizational culture, etc).<br>• Caregivers FIRST Post-Implementation Staff Survey: Quantitative measures to assess teams' ability to successfully implement an evidence-based program (e.g., team size and composition, organizational culture, etc).<br>• Caregivers FIRST Pre-Implementation Staff Interview Guide: Qualitative measures to assess teams' ability to successfully implement an evidence-based program (e.g., team size and composition, organizational culture, etc).<br>• Caregivers FIRST Post-Implementation Staff Interview Guide: Qualitative measures to assess teams' ability to successfully implement an evidence-based program (e.g., team size and composition, organizational culture, etc).<br>• Caregivers FIRST Site-reported EBP Adaptations Form (Baseline, 6-month, and 10-month): This form will be sent to the point of contact at each site to complete at specified timepoints. The form will be used to document site-specific adaptations that are made to the program.
1. Protocol updates:<br>• Pgs. 12-13; 26: Group PT subproject<br>o Refined implementation activities and used "enroll" language vs. "initiate" for implementation outcomes and analysis.<br>• Pg 14. STRIDE subproject<br>o Refined implementation activities and implementation outcomes to specify general medicine patients within month 5 or 6 from study start date.<br>• Pg. 16: Caregivers FIRST subproject<br>o Refined adoption definition to further specify low enrollment (4 or less caregivers trained).<br><br>2. Amendment to Caregivers FIRST subproject data collection instrument:<br>• Caregivers FIRST Pre-Implementation Staff Survey: Revised challenging and helpful implementation factors questions that had "patient" awareness responses to "patient/caregiver".<br>• Caregivers FIRST Post-Implementation Staff Survey: Revised demographics section to match the pre-implementation survey; editorial change to remove references to STRIDE program.

1. Protocol updates:<br>• Pgs. 17-18: Updates to Caregivers FIRST recruitment and randomization strategy, shifting to a more pragmatic approach approved by operational partners to select from nonresponding sites distributed across VISNs with sites systematically selected to approach for enrollment based on site complexity level and lowest implementation activity within a VISN.<br>• Pgs. 14: Updates to STRIDE recruitment strategy to use Microsoft Identity Manager to create a list of potential staff by job title to approach for recruiting their site.<br>2. Amendment to Caregivers FIRST subproject data collection instrument:<br>• Caregivers FIRST 6-month and 10-month adaptations forms: Updated first question to remove references to wards since program is not being implemented on wards.<br>3. Amendment Group PT subproject data collection instruments:<br>• Group PT baseline adaptations form: General updates to language, removal of BCA questions, addition of program staff selection, modification of window the form is open.<br>• Group PT 6-month adaptations form: General updates to language, change to all instances of Group PT Diffusion Network to Group PT Office Hours, and modification of window the form is open.<br>• Group PT 12-month adaptations form: General updates to language, change to all instances of Group PT Diffusion Network to Group PT Office Hours, and modification of window the form is open.<br>• Group PT Pre-implementation survey: minor adjustment to introduction language.<br>• Group PT Post-implementation survey: minor adjustment to introduction language and revision to instances of Diffusion Network in the answer choices.<br>• Group PT Post-implementation interview guide: general updates to language.
1. Protocol updates:<br>• Pg. 17: Updates to Caregivers FIRST 1) total enrolled sites for study, 2) Enhanced REP (enREP) implementation strategy including fielding a needs assessment survey to randomized sites to inform enREP facilitation, 3) updates to randomization procedures for enrolled sites, and 4) refinements to the following implementation outcomes: adoption, penetration, and fidelity.<br>2. NEW data collection instrument:<br>• Caregivers FIRST Enhanced REP Needs Assessment: New instrument for sites randomized to receive enREP.<br>3. Amendment to Caregivers FIRST subproject data collection instrument:<br>• Caregivers FIRST 10-month adaptations form: Simple admin change – updated title that our data collection happening at 12 months vs. 10 months.<br>• Caregivers FIRST post implementation survey: Updated that our data collection is happening at 12 months vs. 10 months; updated that EnREP questions for those randomized sites will be administered at 6 and 12 months; added today's date field to questionnaire.<br>4. Amendment Group PT subproject data collection instruments:<br>• Group PT baseline adaptations form: minor adjustment to introduction language.<br>• Group PT Pre-implementation survey: minor adjustment to introduction language.
1. Protocol updates: Updates to Enhanced REP (enREP) procedures that apply to all projects, as well as specific changes to the enREP procedures for the STRIDE subproject.<br>2. Update to Caregivers FIRST subproject data collection instrument:<br>• CaregiversFIRST Post-imp interview guide_2334_AMD10_20220603: This interview guide has already been submitted and approved. It will be used at baseline and 6 months for CaregiversFIRST. Previously it was only to be used at 6 months.<br>• CaregiversFIRST Pre-imp interview guide_2334_AMD6_20220120_OLD: This interview guide has already been submitted and approved. It will no longer be used for data collection for CaregiversFIRST.

1. Protocol updates: Updates to STRIDE's recruitment procedures that include removing the cap on the number of sites included in each cohort. Updating Group PT's strategy to boost staff survey responses by way of a raffle.<br>2. Group PT Data Collection Instrument Updates: minor, editorial changes to reflect updates and survey set-up in REDCap.
1. Protocol updates: Updates to STRIDE's recruitment procedures that include removing the cap on the number of sites included in the study and minor updates to the enREP language.
The purpose of this submission (AMD 13) is to (1) provide updates to the post-implementation qualitative interview guides for STRIDE and Caregivers FIRST, (2) update server locations for study data storage, and (3) Update Group PT inclusion criteria, and (4) revise the adoption benchmark definition for Group PT.
Continuing Review
The purpose of this submission (AMD 14) is to provide updates to the post-implementation survey for Caregivers FIRST and.
The purpose of this submission is to provide updates to the contact information located in the Group PT Data collection instruments
The purpose of this submission is to revise data collection instruments for Group PT subproject and extend the time that patient outcomes are tracked for sites implementing Group PT.
The purpose of this submission is to add an additional storage location (VA BOX) for our project data
Continuing Review
The purpose of this submission is to update subproject aims, timeframes, outcome definitions (STRIDE and Caregivers FIRST), and analytical approaches (Caregivers FIRST only) as well as the location of our SAS server location (pg.32).
We are reporting a privacy and information security incident (unencrypted transmission of PII).
Added RHF and estimated # subjects for STRIDE & Caregivers FIRST
Updated HIPAA waiver and STRIDE effectiveness estimates
Continuing Review

Background and Significance

Health-related functional impairments often lead to disability, which is a significant driver of health care utilization and costs. Nearly 5 million Veterans currently live with a service-connected disability, and close to $400B is attributed to disability-associated healthcare annually in the U.S. This cost extends beyond the Veteran and also impacts their support system. Approximately 5.5 million Veterans benefit from informal care, that is, regular assistance in the home provided by a family member or friend, for support that can help stave off disability. However, many family caregivers report feeling unprepared and unsupported in their caregiving role. Drawing from the International Classification of Functioning, Disability and Health (ICF) to identify points of vulnerability along the continuum of disability, Function QUERI addresses an urgent need within VA for dissemination of EBPs to address known stressors (e.g. health conditions, hospitalizations) and contextual factors (e.g. inadequate caregiver support) that negatively influence Veterans' functional ability and independence.

Function QUERI's clinical programs were selected on the basis of alignment with national VA strategic goals, those of our operational and clinical partners, and potential impact for Veterans. The underlying clinical framework is the International Classification of Functioning, Disability and Health (ICF, Fig. 1). An individual's functional status is dynamic, with stressors such as hospitalization or development of a symptomatic chronic illness being important tipping points into disability.[1] Each EBP directly addresses known stressors and/or contextual factors that influence functional ability and independence. Project 1 examines implementation and effectiveness of Group Physical Therapy (**Group PT**) for knee osteoarthritis (OA; health condition), a leading cause of pain and disability among VA healthcare users.[2] Many of the negative effects of knee OA can be successfully mitigated through PT, a recommended core component of treatment;[3,4] however, many VA healthcare users with knee OA do not receive PT, and a key barrier is the high demand for PT services in the VA. To address this gap in access, we previously conducted a randomized controlled trial (RCT) comparing group-based PT vs. traditional individual PT for knee OA. The Group PT program consisted of 6 sessions, led by a physical therapist, that focused on evidence-based, functional exercises for individuals with knee OA, along with individual tailoring and guidance on progression and delivery of specific didactic content (e.g., activity pacing and managing pain with exercise). Group PT resulted in equivalent or greater improvements in pain and functional outcomes compared with traditional PT, which is important because the group-based model provides care to more Veterans with fewer staffing resources, thereby increasing access and efficiency.[5] Project 2 compares implementation strategies for large scale spread of **STRIDE**, a supervised walking program for hospitalized older Veterans. STRIDE focuses on maintaining musculoskeletal strength and mobility during hospitalization (environmental factor), a highly vulnerable time for development of disability.[6] STRIDE is designed for patients aged $\geq$ 60 years and consists of a one-time gait and balance assessment conducted by a PT, followed by daily walks supervised by a mobility assistant for the duration of the hospital stay. Clinical demonstration of STRIDE conducted at the Durham VA resulted in a greater likelihood of discharge to home than to skilled nursing or rehabilitation among STRIDE participants compared to clinically similar patients receiving usual care (92 vs 74%, $p = 0.007$).[7] Based on this experience, the cumulative evidence of the positive impact of early mobility interventions,[8-15] and successful spread to other hospitals,[16,17] STRIDE has the potential to become a system-wide approach to address hospital-associated disability in VA. Project 3 examines implementation of **Caregivers FIRST** (Caregivers Finding Important Resources, Support, and Training), an evidence-based skills training program for caregivers of Veterans with cognitive and/or functional limitations. Caregivers FIRST promotes Veteran function and independence through caregiver skill training and support (personal factors) in a series of 4 proactive group classes to help general caregivers build self-care and psychological coping, health system navigation, and hands-on clinical skills. The RCT of Caregivers FIRST

(formerly HI-FIVES) showed sustained improvements in a key quality metric, caregivers' and patients' experience of care.[18] Based on these results and persistent gaps in caregiver training, VA's Caregiver Support Program is partnering with Function QUERI to plan and conduct a national rollout of the program.

Function QUERI Results to Date

The current proposal builds upon and extends work that we began with initial funding of Function QUERI in October 2016. We will continue our focus on the same 3 clinical programs – Group PT, STRIDE, and Caregivers FIRST - because of their importance to VA and Veterans, relevance to our theme, and the opportunity and capability to **move each of them to the next phase of implementation**. Group PT was implemented at one site as a successful QI initiative; the next step is to implement and evaluate in a diverse group of VAMCs. STRIDE and Caregivers FIRST are each being implemented in stepped wedge cluster randomized trials (CRTs; n=8 VAMCs for each trial) which are ongoing. Although full trial results are not yet available, these EBPs have achieved substantial implementation momentum with national program offices endorsing further spread. STRIDE was named a Gold Status Practice in the 2018 VA Diffusion of Excellence Shark Tank competition and in 2019 endorsed by Dr. Carolyn Clancy as one of 3 EBPs in this cohort that should be considered for implementation at all VAMCs. VA's Caregiver Support Program (CSP) has selected Caregivers FIRST for national rollout to all VAMCs to extend the portfolio of services available to caregivers of Veterans as mandated in the MISSION Act. While this endorsement is supportive of implementation, we know that adoption of EBPs rarely happens automatically even when there is policy and stakeholder support.[19] An active approach to implementation is critical to ensure uptake and use of these three EBPs in routine clinical practice.

In the current proposal, each Function QUERI project has unique features based on EBP implementation phase and priorities of VA partners but all are designed to address a common challenge encountered in our work to date – **the need to advance our understanding of how to deliver the right dose of support to implement and sustain new programs**. For example, in our ongoing work, we supported 8 VAMCs in implementing STRIDE using Replicating Effective Programs (REP) with external facilitation as implementation strategies. Although sites responded positively to support provided by the Function QUERI team, **this approach was too time-intensive (approximately 100-140 hours/site) to be replicated on a national scale**.[17] In addition, at the request of VISN 6, Function QUERI supported STRIDE implementation without facilitation in 6 other sites, and this experience provided clear evidence that some, but not all, sites are able to launch EBPs with access to program materials and minimal technical assistance.[16] Similarly, one VAMC successfully implemented Caregivers FIRST independently, using only a toolkit. **Therefore, our new Function QUERI projects will test approaches to monitor site implementation progress and deploy higher intensity strategies for sites that have not met implementation adoption benchmarks.**

Our initial 3 years have demonstrated our ability to conduct this work successfully. To date, we have supported implementation of Group PT, STRIDE, and Caregivers FIRST across 12 VISNs with programs reaching 1700+ Veterans and caregivers and 700+ providers trained. We have reported our findings in 10 journal publications[16-18,20-26] and more than 79 presentations, including briefings to VA leaders, presentations at scientific meetings, and HSR&D cyberseminars. We will leverage tools, resources and expertise from our current work and have proposed projects with independent aims that are not contingent on the results of ongoing studies. We have not yet asked sites to sign formal participation agreements; however, we have confidence in our ability to enroll our target numbers based on our success recruiting sites to date, expressed interest from VAMCs across the country and strong commitments from VISN and program office partners to assist in this effort.

Design

All three implementation projects are designed to inform quality improvement efforts, through supporting implementation and evaluation of the three EBPs on a national scale, assessing their impact on quality metrics, and advancing application of implementation strategies in the VA (see QI certification letters from national program partner offices in the *Appendix*). The projects will involve use of secondary and primary VA data collected using assessments that are part of routine care and/or clinical management. The projects will be collecting information that is designed for quality improvement initiatives, as described in Program Guide 1200.21, for the purposes of program implementation/evaluation. In addition, the Function QUERI team will partner with VA leaders to conduct rapid, high-quality implementation and evaluation projects (Rapid Response Team) and train the next generation of implementation scientists to become leaders and innovators in VA implementation science and quality improvement (Mentoring Core).

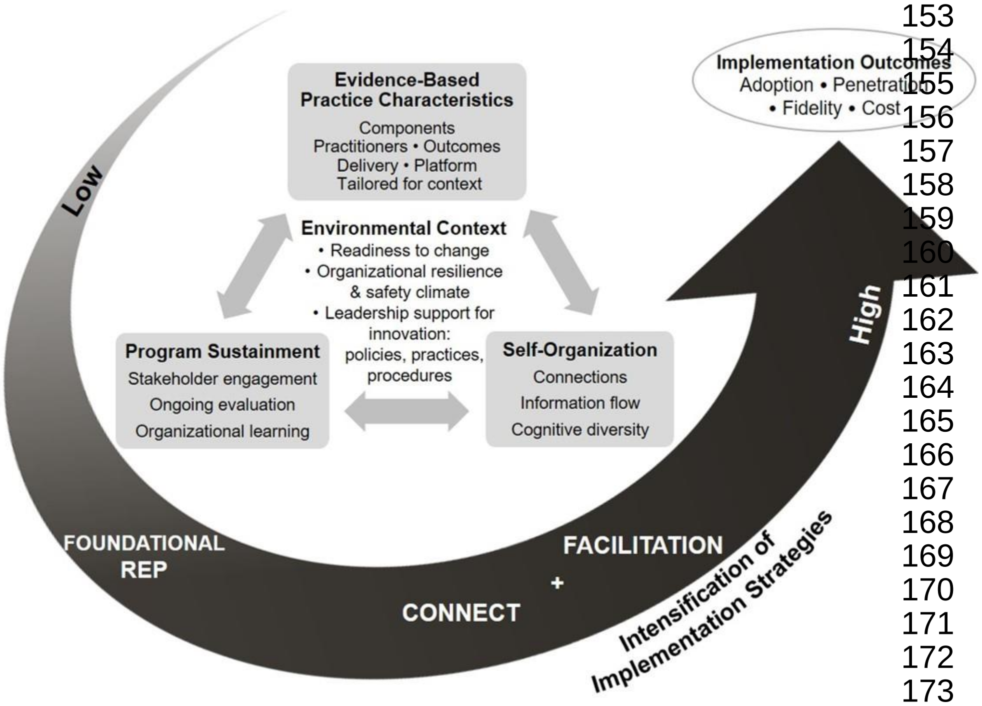

Figure 2. Function QUERI Implementation Framework

Implementation Core

The Implementation Core serves as an essential bridge across all Function QUERI projects. Guided by the QUERI Implementation Roadmap[27] and the Dynamic Sustainability Framework,[28] the Core operates under the assumption that implementation is a dynamic and iterative process in which greater sustainment is achieved by adapting EBPs for fit, engaging stakeholders, and evaluating impact on Veterans and the health system. Specifically, the Core will support and create synergies across Function QUERI's program of work by leading efforts to develop and deliver implementation strategies (Aim 1), providing guidance on a common set of measures to evaluate implementation and impact of EBPs (Aim 2), synthesizing findings on approaches to intensify implementation support to inform plans for organizational sustainment and transition of EBP ownership to stakeholders (Aim 3), and contributing expertise in implementation science for time-sensitive requests from partners and through mentoring (Aims 4, 5).

Implementation Framework. We have constructed an overarching framework (Fig. 2) for conducting and evaluating implementation activities that draws from the Dynamic Sustainability Framework,[28] complexity science,[29] and Proctor's taxonomy of implementation outcomes.[30] The model posits that implementation of new programs depends on factors related to EBP characteristics and an interaction of these factors with a site's environmental context, processes to support program sustainment, and supporting teams' capacity to self-organize for optimal problem-solving. Specifically, we anticipate that organizations with a high degree of readiness to change, safety climate, and resilience ethos will be more successful with implementation of the three EBPs. Because of variation in context and organizational characteristics, we propose that low intensity implementation support that promotes adapting EBPs for context and provides tools for ongoing EBP evaluation (defined as foundational REP), will be sufficient for some but not all sites to successfully incorporate new EBPs into routine practice. Further, we posit that monitoring sites' progress and adding, for sites with low adoption, higher intensity strategies that directly influence teams' capacity and skills to effectively self-organize and problem-solve will lead to higher implementation adoption, penetration, fidelity, and value.

Implementation Strategies. Guided by our framework and our prior Function QUERI work, we will use **Replicating Effective Programs (REP)** as the foundational approach supporting implementation of each EBP. REP has been described both as an implementation framework[31] and strategy.[32] Herein we refer to REP as an implementation strategy because it will be an integrated bundle of discrete activities selected to address identified barriers to implementation success.[33] REP was used in the prior Function QUERI projects because it addresses a major barrier to adoption of new clinical programs for Veterans at risk for functional decline: limited clinical resources. REP addresses this barrier by promoting flexibility and potential adaptation in the delivery of clinical programs so that VAMCs can select approaches that best fit local conditions and resources. Our approach to monitoring and understanding adaptations will be guided by the FRAME, a framework for reporting adaptations to EBPs.[34] REP has been empirically tested and validated through RCTs and shown to be effective in promoting uptake and fidelity of clinical interventions in various healthcare organizations, including VA.[31,32,35,36]

Foundational REP Activities. REP is designed for roll-out of new programs through four phases: pre-condition, pre-implementation, implementation, and maintenance, with a combination of standardized activities across each phase. Pre-condition activities, such as identifying gaps in clinical care and the EBPs to fill these gaps, and pre-implementation activities, such as identifying barriers to implementation, occurred during our prior Function QUERI work. Based on lessons learned and input from partners, foundational REP implementation activities will include 5 common elements across all EBPs that were developed and tested in our prior Function QUERI work:

1) **Stakeholder engagement**; each project will convene a panel of national and VISN stakeholders prior to kickoff and engage continually with our Technical Expert Panel and Veteran engagement panel;
2) **EBP toolkits** with a) standardized program materials to help clinical staff implement the EBP, including recorded webinars and b) guidance on core and modifiable components of each EBP and options for customization;
3) **SharePoint** for access to electronic health record (EHR) CPRS/Cerner templates, clinical program training materials, and standardized materials to facilitate monitoring sites' progress;
4) **Data dashboards** including from the VA Support Service Center (VSSC) to assist sites with tracking their own data; and
5) **Diffusion Networks**, which are a blended strategy to support both initial implementation and sustainment. The goals are to capture and share local knowledge and create a

collaborative environment for peer-to-peer sharing of experiences and best practices to support implementation via monthly teleconferences. Function QUERI launched a Diffusion Network for STRIDE in 2018 and has found it to be a very effective means for providing technical assistance and ongoing consultation, key components of REP.

The final phase of REP is maintenance, which will include development of implementation plans for national and regional partners so that they can ultimately own and sustain the implementation process for each EBP over time. REP has many advantages as an implementation strategy; there are limitations to its use as well. For example, REP is not designed to address differences in organizational readiness or resilience (i.e., capacity), which may limit the ability of some sites to adopt a new EBP. **Function QUERI addresses this potential limitation by supplementing REP with higher intensity implementation strategies for sites not meeting benchmarks for adoption of each EBP.**

Enhanced REP (enREP) Activities. EnREP begins with the same activities as foundational REP. Sites that were initially randomized to the enREP group and do not meet EBP-specific *a priori* benchmarks reflecting adoption within 6 months will continue with foundational REP and will receive higher intensity support for a period of 4-6 months (enREP). The higher intensity support will consist of tailored sessions including a barrier/needs assessment and *facilitation*, a process of interactive problem solving and support that occurs in a context of a supportive interpersonal relationship.[37] Sites will participate in a minimum of 3 to 4 sessions with additional sessions available upon a site's request. Sites will be asked to self-identify and rank perceived barriers to implementation. Facilitators will follow an evidence-informed table specifying identified barriers to implementation, along with potential aligned strategies to overcome the barriers, and examples in the context of the EBP. Facilitation will be provided by Function QUERI team members who are experts in implementation and have close collaborations with EBP content experts (for example, the Durham VA physical therapist who leads Group PT classes). Each call will be attended by additional members of the research team who will listen and take structured notes regarding call fidelity monitoring and implementation tracking. Facilitators' actions will depend on each site's needs and clinical context. As a specific example, if a site reports problems with documenting information in the EHR, then the facilitator could forge connections between a CAC working with the research team and a CAC at a site. Based on our current work, we anticipate facilitators will help sites engage facility leadership, provide resource materials, identify and address barriers to program launch, and support marketing efforts. EnREP may include *CONNECT*, a complexity science-based bundle of interaction-oriented activities designed to supplement implementation efforts by promoting team function and readiness for change.[22] CONNECT may be included as a component of implementation intensification because of its specific focus on building skills to optimize team function, which complements activities of facilitation.[22,38-40] All three EBPs rely, in different ways, on interactions across different types of providers and clinical services, making team function and communication essential for successful implementation. Therefore, we propose that CONNECT will be specifically relevant for sites that do not exhibit successful EBP adoption and express challenges around team communication. Our prior Function QUERI work found that CONNECT was viewed as helpful for understanding communication patterns and breakdowns across service lines and facilitating cross-service interactions, but that it could be improved by identifying key players to facilitate effective communication and engaging team members throughout the implementation process. Based on these findings, CONNECT can be tailored for each EBP and challenges of individual sites, utilizing a menu of activities we have experience with: relationship mapping of communication patterns, identification of key cross-service communicators, strategies for creative, ongoing problem solving, and mentorship to sustain new interaction behaviors.[38]

Implementation Evaluation. We will use mixed-method designs for two distinct but complementary purposes in the overall evaluation of each project. We describe our mixed-method approaches here, as we will use similar techniques across all projects. Data sources will include 1) EHR data accessed through CDW and JLV; 2) implementation process data such as site-reported EBP adaptations, and field and call notes including polls (e.g., Webex or Poll Everywhere); and 3) quantitative surveys and semi-structured interviews completed twice, in the: a) pre-implementation phase at baseline; and b) post-implementation phase, which is 10-18 months post-implementation depending on the EBP (Fig. 3).

First, each project will employ an **explanatory sequential mixed method design that includes a cluster-randomized trial and qualitative data collection and analysis**[41] (QUAN → *qual*) to evaluate implementation of each EBP (Fig. 3). The quantitative component will indicate effect of study arm on implementation outcomes; specific quantitative analyses are described in each individual project section. The qualitative component will shed light on sites' experiences with implementation activities and perceptions of intensification in order to address a common question across projects*: How do sites experience implementation?* We will use 30-minute individual or group semi-structured telephone interviews with key informants to elicit detailed description of the facilitators and barriers that affected their implementation of the EBPs. Specifically, we will ask about detailed activities their site used to implement the EBPs and any additional strategies that they developed, probing for details based on Proctor's criteria for specifying and reporting strategies (e.g., actor, action, target, temporality, frequency).[42] We will ask representatives from each site to describe conditions that have facilitated and impeded implementation. We will additionally interview leaders who are more distal to the day-to-day operations (e.g., chief of staff, facility director) about conditions that they view as relevant for implementing the EBPs to enhance our understanding from different vantage points. Interviews will be audio recorded and transcribed. We will use directed content analysis[43] that includes both *a priori* labels to mark REP activities and implementation strategies based on the Expert Recommendations for Implementing Change (ERIC) typology[37,44] (e.g., "engagement with toolkit") and data derived labels to reflect respondents' description of their experience with barriers to implementation. We will then summarize the coded data in a framework matrix[45] to compare reports of implementation strategies and barriers across sites. The rows of the matrix will reflect coded implementation strategies and the columns will reflect whether or not responses are from foundational REP sites and implementation outcomes for each site. Summaries of coded data within each matrix cell will describe the implementation strategy; we will assign valence (+ or -) to indicate whether data, based on aggregate of individual interviews from each site according to our interpretation of respondents' reports, manifest as facilitators or barriers to implementation. At least two researchers will independently code transcripts and assign valence to data and then will meet to compare codes and resolve discrepancies. The qualitative researchers will meet with the project director and other team members to review the matrix and identify patterns between implementation approaches and sites with lower versus higher implementation outcomes. Qualitative data will be organized and analyzed using the Atlas.ti or NVIVO qualitative software packages currently licensed and approved for use on VA computers. All data will be saved to secure project folders.

Second, we will employ a **qualitatively driven convergent design** (QUAL + *quan)* in which pre-implementation qualitative data are collected at baseline in parallel with quantitative data in order to explore and understand pre-implementation contextual factors related to differences in site-level adoption, addressing the question: *What baseline organizational and implementation team characteristics are associated with sites that don't meet adoption benchmarks*? All projects will use similar approaches to understand organizational readiness to change, organizational resilience, and other common measures. Data sources will include pre-

implementation quantitative surveys and qualitative interviews at baseline to understand organizational and contextual factors and EHR data to define adoption. Key informants will include members of the implementation team as well as organizational leaders (e.g., chief of staff, facility director). In our ongoing Function QUERI work our richest understanding of context has been through qualitative inquiry, and we will use quantitative data collected via surveys administered to site hospital and service-line leadership and EBP implementation team members as an added dimension for understanding qualitative findings.[41,46] In order to increase survey participation, we may offer incentives such as an entry into a raffle for small prizes.

Common measures. Consistent with our framework (Fig. 2) we assert that team factors, environmental context and implementation processes including EBP adaptations are critical factors in influencing implementation outcomes. Our ongoing Function QUERI work has demonstrated heterogeneity in extent of adoption of EBPs designed to promote function and independence. An important component of our proposed work is to explore potential pre-implementation contextual contributors to this heterogeneity. Below we provide an overview of domains and example quantitative measures that will be used across projects. Data from survey measures will be integrated with qualitative data as described above.

Implementation team processes, functioning and self-organization. Team measures will be collected via pre-implementation baseline survey. These measures will include an assessment of team size and team composition. These measures were also assessed in our prior Function QUERI work and determined to be salient factors in teams' ability to successfully implement the EBPs. Other measures will include organizational culture, technological availability,[47] and participation in decision making.[48]

Environmental context. We will evaluate environmental context using validated measures that address the following constructs: organizational resilience,[49] organizational readiness to change,[50] and safety culture.[51] Organizational resilience is a new area of focus because it emerged as a central factor that was much broader than the role of team processes. Organizational resilience is the capacity of an organization to anticipate, prepare for, respond, and adapt to both incremental changes and sudden disruptions in order to survive and thrive.[52] We will assess resilience quantitatively using a validated measure that assesses active problem-solving, team efficacy, confident sense-making, and bricolage.[49] Organizational readiness to change addresses organizational members' shared resolve to implement a change (change commitment) and shared belief in their collective capability to make a change (change efficacy).[50,53] Survey responses will include a five-point Likert scale (*e.g*., ranging from 0 = 'not at all confident' or 'don't know', to 4 = 'very confident'). We will perform descriptive statistical analyses to calculate percent, mean and standard deviation for individual measures and scales. The VA REDCap survey platform will be used within the VA firewall to survey key informants, who will be sent a VA email inviting them to participate.

Implementation process measures including adaptation. EBP adaptations will be reported using Wiltsey Stirman's FRAME, which provides a standardized way to track modifications and facilitate continual monitoring of the fit of the EBP within organizations.[34] FRAME specifies the importance of reporting aspects of adaptations that are often overlooked such as (1) when and how in the implementation process a modification was made, (2) whether the modification was proactive or reactive, and (3) who determined that the modification should be made. STRIDE will use FRAME to characterize adaptations among existing programs to evaluate associations with greater implementation success and will use these data to make recommendations to new sites. In addition, all EBPs will use FRAME as a reporting tool to track adaptations in new projects to provide insight into the implementation process.

Implementation Outcomes. Following the taxonomy defined by Proctor and colleagues[30] and given the overall goals of Function QUERI and our operational partners, we will focus on 4 main types of implementation outcomes: **adoption**, **penetration**, **fidelity**, and **cost**. Adoption will be operationalized as actual uptake of the EBP, with specific definitions described below for each EBP. Penetration is the level of integration of the program within a VAMC's relevant clinical units. This will be assessed through quantitative data obtained through the EHR (CDW and JLV) and through qualitative interviews. Finally, using procedures developed in prior work, fidelity to the EBPs and to the implementation strategies will be assessed through surveys, EHR data and qualitative interviews. Costs will be assessed using implementation process data and VHA salary and managerial cost accounting data.

Analyses of business case/value proposition. Business Case Analysis (BCA) will frame affordability to VA for each EBP.[54] We will draw on methods established in the original Function QUERI.[55] Two types of costs will be collected for each site using a standardized method to track implementation activities: (1) clinical delivery team costs incurred; and (2) implementation strategy costs incurred. Unlike in a cost-effectiveness analysis evaluating two distinct treatments, we will not evaluate health care costs because all individual participants will receive the same clinical treatment regardless of a site's implementation strategy. First, for clinical delivery team costs, we will measure personnel time and labor costs associated with preparing for and delivering the clinical programs using micro-costing and periodic time studies. Labor costs will be valued using VA Human Resources salary data and program-related durable medical equipment costs will be valued at their purchase price (e.g. walkers, stop watches). Second, we will track implementation strategy costs, including training time, training materials, and time of both the Function QUERI team and site team members outside of program delivery time. Value will be defined in light of the implementation and quality outcomes of key interest for each EBP. The Implementation Core will work with each EBP team to refine which a priori outcomes will be used to assess value.

The base-case BCA will use a decision tree to compare the expected value for costs and outcomes between treatment arms. In addition, we will use one-way and probabilistic sensitivity analysis to simulate likely outcomes in the context of distributions informed by the trial data as well as prior evidence for the EBP. By modeling plausible scenarios, the decision model will allow us to communicate a practical range of estimates to sites and operational partners rather than relying on statistical significance. Monte Carlo probabilistic sensitivity analysis will be used to incorporate measured uncertainty in the estimates for all outcomes and generate probabilities for exceeding VA thresholds for value. We will work with our stakeholder partners to establish thresholds.[56] For each EBP we will report costs by arm for the base case BCA and for different assumptions, along with what conditions would need to be met to be within VA thresholds for affordability/value using the Monte Carlo results.

Effectiveness outcomes/Quality metrics. For each EBP we will assess impact on quality of care goals outlined in the VHA Medical Center and Network Director Performance Plan, MISSION Act and other meaningful measures. Measures have been selected based on a) our clinical theme of function and independence and b) outcomes and quality metrics we expect to be positively affected by each EBP (e.g. SAIL metrics such as hospital length of stay).

Leadership. Dr. Leah Zullig will serve as Director of the Implementation Core. She has formal VA and NCI-funded implementation science training, teaches implementation science courses at Duke University and the University of North Carolina, and directs INTERACT, the Implementation Science Research Collaborative, at the Duke Department of Population Health

Sciences. Dr. Zullig has a strong, consistent history of funding for implementation science projects in both leadership and supportive roles.

Implementation Projects

Overview. Function QUERI will support implementation of 3 EBPs in VAMCs across the country. All projects utilize a type III effectiveness-implementation hybrid design framework, in that the primary goal is to compare implementation strategies while also observing/gathering information on the clinical intervention and related outcomes.[57] All projects involve a CRT. Fig. 3 provides an overview of project designs.

Group PT and STRIDE will randomize sites 1:1 to foundational REP or enREP, which consists of adding higher intensity implementation support for VAMCs that do not meet adoption goals at 6 months nor sustainment goals at months 8 and 9 (for STRIDE and GPT, respectively). The Caregiver FIRST design is slightly different to take advantage of the fact that CSP requested national implementation to address unmet needs for training and to support the expansion of general caregiver services required by the MISSION Act. Following Caregiver FIRST roll-out at all 150 medical centers, we will enroll a subset of sites that did not meet a priori adoption benchmarks at 6 months (n=24) and randomize them 1:1 to continue foundational REP or receive higher intensity implementation support. A run-in design is critical for this project for efficient use of resources because we cannot forecast which sites will be able to launch the mandatory EBP independently and which will need a more intensive approach to adopt the EBP. All projects use a common set of foundational REP activities and higher intensity strategies. Primary outcomes will be assessed at project specific time points between 10-18 months from the beginning of foundational REP, based on previous experience of how long it takes to implement each EBP.

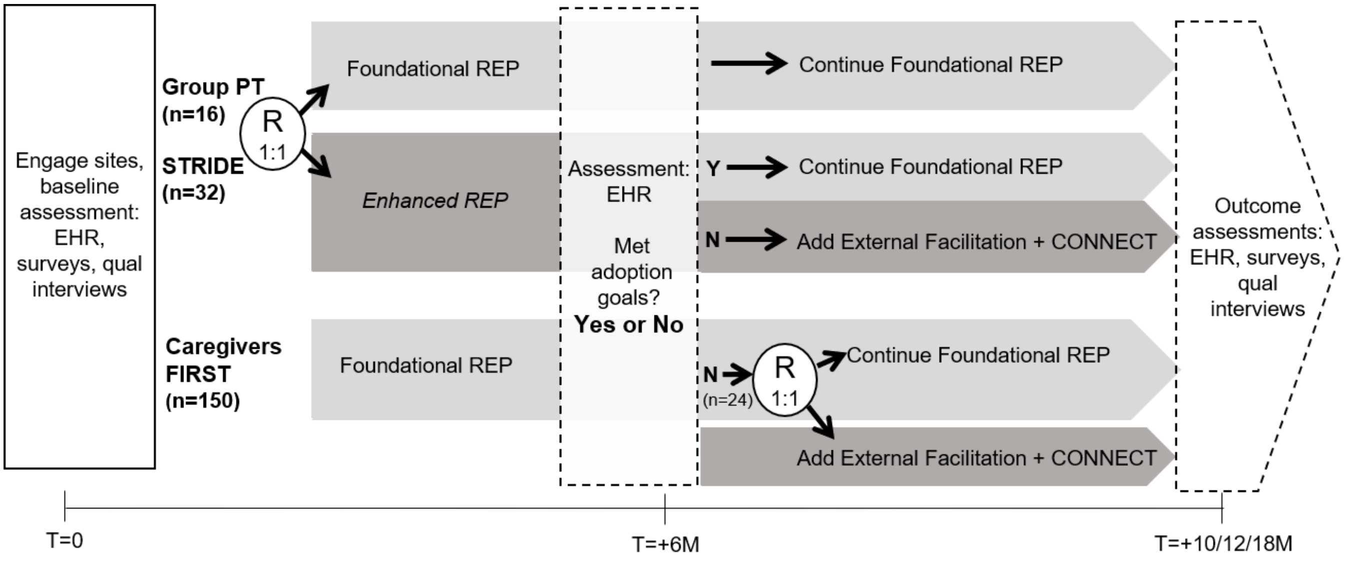

Figure 3. Function QUERI Project Design Overview

Implementation Project 1: Group PT for Knee OA

**Objective**	Improve access to PT to Veterans with knee osteoarthritis by delivering PT in a group setting
**Evidence**	Allen et al. Group Versus Individual Physical Therapy for Veterans With Knee Osteoarthritis: Randomized Clinical Trial. *Phys Ther* 2016, 96:597-608.
**Primary Partners**	VA Physical Medicine & Rehabilitation Program

Urgency of improving functional outcomes in Veterans with knee OA and relationship to ICF. Knee OA is a common and often disabling health condition that affects 14 million people in the US,[58] and rates are about twice as high among Veterans than the general population.[59-61] Forty-three percent of VA healthcare users report a diagnosis of arthritis (primarily OA), and of these, 63% report activity limitations due to joint symptoms.[62] The prevalence of OA is expected to rise dramatically over the next several decades,[63] and this will place increasing demand on the VA to provide core, evidence-based components of knee OA care, including PT. Pain and functional limitations are key issues among Veterans with knee OA, and these in turn lead to negative impacts across many other health outcomes.[64-68] Notably, recent research indicates knee OA is independently associated with incident cardiovascular events and mortality risk, at least partly through a pathway of functional decline.[69,70]

Project Goal. The overall goal of this project is to implement, evaluate and sustain Group PT in 16 VA sites. We will address the following specific aims:

<u>Specific Aim 1</u>**:** Develop a scalable approach to implement and sustain Group PT.

Key Questions: What are stakeholder perspectives on key resources required for EBP delivery and sustainability? What are appropriate adaptations including incorporation of telehealth within Group PT?

<u>Specific Aim 2</u>**:** Evaluate implementation of Group PT with foundational REP versus enREP.

Key Questions: Are there differences in implementation outcomes (adoption, penetration, and fidelity) at 6 and 12 months between arms? What are effectiveness outcomes (function, pain) at implementing sites? How do sites experience implementation strategies in each arm? What baseline organizational characteristics are associated with sites that don't meet adoption benchmarks?

Study design and methods. The Function QUERI team will convene a panel of stakeholders (e.g., clinicians and operations partners) to achieve the following <u>Aim 1</u> activities: 1) Develop and refine tools and processes to be used in foundational REP activities (e.g. data reports, Office hour calls) and 2) Propose Group PT adaptations that sites may choose to enhance delivery and fit within their context (e.g. "rolling" entry to the program vs. starting new cohorts together; inclusion of a physical therapy assistant in leading group exercises vs. other staff; and patient participation via telehealth vs. in-person). For the CRT addressing <u>Aim 2</u>, 16 VAMCs will be randomized (1:1) to either foundational REP or enREP (Fig. 3). Participating sites must meet the following criteria: 1) Clinical personnel on staff to conduct initial evaluations and lead group classes (e,g, physical therapist, PT assistant, kinesiotherapist): this should include at least 1 primary person and 1 back-up person to cover all aspects of program delivery, 2) Offer outpatient PT service, and 3) Space to conduct group sessions (if implementing in-person Group PT classes). Sites that are currently offering a group class specifically for knee OA will not be eligible to participate. Because rural sites often have fewer PTs and therefore a particular need for strategies to maximize efficiency of delivering these services, at least 25% of sites will be rural. Specifically, we will use the ORH rurality calculator, which pulls data from the Current Enrollment Cube, using closest facility or county/zip code level data to determine percent of rural veterans served by a facility We will use a target threshold of ≥50% rural/highly rural Veterans served by a facility when recruiting sites. If we cannot reach our benchmark (25% of sites) using this threshold, we will then target sites at which 40-50% of Veterans served are

classified as rural or highly rural. Using methods that have been successful in our prior Function QUERI work, and in partnership with the Physical Medicine & Rehabilitation Service and VISN 6, we will invite participation in the Group PT project in national and regional forums, e.g. monthly PM&R and Chiefs of Staff calls. Randomization will be stratified based on the Complexity Level, where sites 1a, 1b, and 1c are one category and all others (2, 3, CBOCs/ Health Care Centers) are the other category (bottom quartile vs. higher; MISSION Act Quality Standard) and rurality, where sites serving ≥50% rural/highly rural Veterans are one category and sites serving <50% rural/highly rural Veterans are another category.

Implementation activities. *Foundational REP* has been described previously. One component of Foundation REP is the data dashboard, which will assist sites with tracking their own data. The FQ team will use CDW data (e.g., # of consults, # of completed evaluations, etc.) to generate a tool to assist sites with quality improvement efforts. Data will be summarized and free of PHI/PII. Reports will be made available through email to the study sites or via the Group PT SharePoint site (access limited to enrolled sites). *EnREP* begins with the same activities as foundational REP. After 6 months, sites randomized to enREP (n=8) that do not meet adoption benchmarks will receive 6 months of intensified implementation support. Adoption will be defined as delivery of Group PT as a clinical service (delivery of at least 1 Group PT class) and enrollment of at least 5 patients within a 6-month period. Patients must attend at least one Group PT class to count towards enrollment metrics. Additionally, sites randomized to enREP that met adoption benchmarks but do not meet the sustainment benchmark (defined as enrolling 15 new patients between months 7-9), will also receive intensified implementation support for the remainder of the study period. Higher intensity strategies will consist of calls between site implementation teams and a Function QUERI facilitator every 2-3 weeks to promote interactive problem solving in the context of a supportive interpersonal relationship. For the quantitative surveys that are completed twice by participating VA staff, (in the pre-implementation phase at baseline and post-implementation phase at 12 months) we will offer a raffle for small prizes ($30-$50) as an incentive to increase survey completion.

Patient sample. Patients will be eligible for the Group PT if they have a clinician diagnosis of symptomatic knee OA. Participants will not be included if they have knee pain from another condition, have substantial fall risk, or have other co-occurring health conditions that would make participation in a group exercise class unsafe.

Outcomes. *Implementation Outcomes*: Implementation **adoption** will be defined as delivery of Group PT as a clinical service (delivery of at least 1 Group PT class) and enrollment of at least 5 patients within a 6-month period. Patients must attend at least one Group PT class to count towards enrollment metrics. **Penetration** (primary implementation outcome) will be defined as the average number of patients enrolling in the EBP on a monthly basis. **Fidelity** will be defined as the average number of sessions attended by patients who enroll in the EBP. We will also assess the number of referrals to the program, along with the proportion of referrals that result in patients enrolling in the program and reasons for failure to enroll. These metrics will be assessed at 6 months and 12 months (primary) following the initiation of the implementation period.

Effectiveness Outcomes/Quality Metrics will be collected by the physical therapist (e.g., via pen and paper or VA approved survey platform such as VA Qualtrics Research) and documented as health factors in the EHR (within a standardized clinic note template developed by the Function QUERI team), during every group PT class (six total). (*Note: Qualtrics is commercially available and the only approved instance for VA use is the VA Qualtrics Research listed in the ATO.*) These patient-level measures focus on functional outcomes and experience of care, in alignment with the CMS Meaningful Measures Framework, including the Patient-Reported Outcomes Measurement Information System (PROMIS), 30-second chair stand,

maximum pain during the chair stand test on a numeric rating scale (0-10) and patient satisfaction.[72-74] We will continue to track patient outcomes beyond the 12-month formal engagement with enrolled sites to monitor continued progress with Group PT delivery. We may also generate additional data reports for sites to support program sustainment. As outlined above (Group PT Implementation Activities), these reports will be free of PHI/PII and made available through email or via the Group PT SharePoint site (access limited).

Analyses. To address the question: *Are there differences in implementation outcomes (adoption, penetration, fidelity) at 6 and 12 months between arms?* We hypothesize that sites randomized to enREP will have superior *implementation outcomes*, including higher **adoption**, **penetration** and **fidelity**, compared with foundational REP sites at 12-months. We will use generalized linear models[75] to examine the effect of foundational vs. enREP on implementation outcomes at 12-months. The main predictors of interest will be Foundational REP vs. enREP and stratification variables for the Complexity Level, where sites 1a, 1b, and 1c are one category and all others (2, 3, CBOCs/ Health Care Centers) are the other category (bottom quartile vs. higher) and Rurality, where sites serving ≥50% rural/highly rural Veterans are one category and sites serving <50% rural/highly rural Veterans are another category. We will examine how implementation outcomes change over time using descriptive methods (e.g. plots, descriptive statistics, subgroups). To address the question: *What are effectiveness outcomes (function, pain) at implementing sites*? We will describe effectiveness/quality outcomes for patients who enroll in the Group PT program, overall and by study arm. We will calculate descriptive statistics for all visits, and change outcomes, among patients who have data from the first and last visits. Qualitative data collection and approaches to data integration are described previously.

Sample size estimate. Sample size calculations were conducted for the implementation outcomes at 12-months. Using a two-sided t-test based on a sample size of 16 sites (8 foundational REP and 8 enREP), a type-1 error rate of 5%, we will have 80% power to detect an effect size difference of 1.5 and 90% power to detect an effect size difference of 1.7 between arms. For the primary penetration outcome, assuming standard deviations ranging from 1.5 to 2.5 based on data of number of new patients per month over a 1-year period of implementation of Group PT in Durham, these effect size differences correspond to differences in mean number of initiators per month ranging from approximately 2.3 to 3.8 between arms for 80% power and 2.6 to 4.3 for 90% power.

Leadership. Dr. Allen is a health services researcher, an exercise scientist, VA HSR&D Research Career Scientist and Associate Director of the Durham HSR&D Center of Innovation to Accelerate Discovery and Practice Transformation (COIN ADAPT). She is an expert in pragmatic and implementation-focused trials of rehabilitation interventions, with an emphasis on functional outcomes.

Implementation Project 2: STRIDE

**Objective**	Reduce preventable harm of hospital-associated disability through a supervised walking program	
**Evidence**	Hastings et al. Assisted early mobility for hospitalized older veterans: preliminary data from the STRIDE program. J Am Geriatr Soc 2014, 62:2180-2184.	
**Primary Partners**	Geriatrics and Extended Care	VHA Diffusion of Excellence

Urgency of addressing hospital-associated disability and relationship to ICF. Hospitalization is a major (environmental) risk factor for development of disability. More than one-third of adults over the age of 70 are discharged from the hospital with a major new disability that was not present before the onset of acute illness.[6] A key contributor to hospital-associated disability is immobility during hospitalization.[76] Although fewer than 5% of patients have physician orders for bed rest, hospitalized older adults spend only about 3% of their time standing or walking.[77] The hazards of bedrest have been recognized for more than 2 decades,[78] but there remains a persistent ‘epidemic of immobility’ in American hospitals.[79]

Project Goal. The overall goal of the project is to implement, evaluate and sustain STRIDE in at least 32 additional VAMCs. We will address the following specific aims:

Specific Aim 1: Develop scalable approaches to implement and sustain STRIDE.

Key questions: What are stakeholder perspectives on key elements of a large-scale dissemination plan? How should the STRIDE clinical program be adapted to optimize sustainability?

Specific Aim 2: Evaluate implementation with foundational REP versus enhanced REP.

Key questions: Are there differences in implementation outcomes (penetration, fidelity) at 10 months (primary), 13, and 16 months between arms? What is the impact on effectiveness outcomes and quality metrics (hospital length of stay or discharge to nursing home) at implementing sites? How do sites experience implementation strategies in each arm? What baseline organizational characteristics are associated with sites that do not meet implementation benchmarks?

Study design and methods

To address Aim 1, first, we will convene a national stakeholder meeting attended by VISN and program office leaders to obtain and incorporate diverse perspectives in planning for large scale dissemination of STRIDE, funded by VHA Diffusion of Excellence. Second, we will use the Wiltsey Stirman FRAME[34] to classify STRIDE adaptations at 17 current sites and examine associations with implementation outcomes. These data will allow us to update existing clinical program materials to help implementing facilities make informed choices about adapting the STRIDE EBP to fit local resources and needs.

In the CRT to address Aim 2 we will randomize sites 1:1 to either foundational REP or enREP (Fig. 3). In partnership with Diffusion of Excellence, we will invite all VISN Directors to encourage medical centers within their region to start STRIDE programs. This approach has worked well within VISN 6, and other VISNs have agreed to participate. Additionally, we will utilize Microsoft Identity Manager to compile a list of professionals by job title who may be willing to implement STRIDE at their facilities. We will reach out to them via email or Microsoft TEAMS to gauge interest. Sites will be enrolled in cohorts with the goal of enrolling at least 32 sites. We plan on conducting a stratified randomization[80-83] with the following site level covariates assessed prior to randomization: facility complexity level (1a complexity vs. all others), general medicine adjusted length of stay (adjusted length of stay ≥ 4.7 days vs < 4.7 days), and whether the hospital has previously attempted to start a mobility program or received resources to start STRIDE (yes to either vs. no).. These characteristics were chosen to represent factors likely to be associated with baseline differences in sites that may affect implementation outcomes-low bandwidth, presence of local innovations, competing priorities and needs alignment.[84] General medicine adjusted length of stay will be captured using the Strategic Analytics for Improvement and Learning Value Model (SAIL) which assess key quality measures as well as overall efficiency at individual VAMCs. Previous experience with mobility programs will be determined by standard intake forms completed by POCs at each hospital asking if (1) anyone at their facility previously tried to start a STRIDE or other mobility program on a general medicine ward in the past five years and (2) if they have received resources or other staffing support from their VISN or a VA program office to start a STRIDE program. **Implementation activities.** *Foundational REP* activities have been described previously. *EnREP* begins with the same activities as foundational REP. After 6 months, sites randomized to enREP that do not meet adoption benchmarks (defined as 5 general medicine patients with a STRIDE walk in months 5-6; assessed at the end of month 6 will receive higher intensity support sessions for 4-5 months (depending on site availability), consisting of the site implementation team and a Function QUERI facilitator. Sessions will be scheduled in collaboration with the site to promote interactive problem solving in the context of a supportive interpersonal relationship. At the completion of the Function QUERI study, all participating STRIDE sites will receive a thank-you email and small gift bags to be shared among team members (e.g., water bottles, lunch box, t-shirts, hats,

etc.) to show appreciation. STRIDE sites will be unaware that they will receive a thank-you gift bag and therefore would not be considered as an incentive for study participation.

Patient sample. Clinical criteria for STRIDE participation at each site will include: Age >= 60, admission for medical illness, community-dwelling (i.e. not in a nursing home), not requiring inpatient physical therapy, ability to follow one-step commands and to ambulate safely and independently.

Outcomes. *Implementation outcomes.* **Penetration** (primary outcome) is defined as the proportion of eligible hospitalizations with at least one documented STRIDE walk. It is not possible to assess all eligibility criteria in the EHR (e.g. able to walk at baseline); thus, with this more inclusive denominator, 100% penetration is not an appropriate goal. Based on preliminary data, we anticipate penetration to range from 0% (no program activity at 10-month outcome assessment) to 40% (estimated maximum achievable based on data from current STRIDE sites). **Fidelity** will be assessed for eligible hospitalizations with at least one documented STRIDE walk as the percentage of eligible hospital days with "full dose" of the program, defined as two or more documented walks or one for more than five minutes. Program **adoption** is defined as ≥ 5 general medicine patients with a STRIDE walk in the EHR within month 5 or 6 from start date with Function QUERI. We will assess implementation outcomes at 10 months (primary), as all current STRIDE sites have launched within this time frame, and also at 13, and 16 months to describe trajectories and assess sustainment, given that we have observed the dynamic nature of STRIDE program activity at some sites.

Effectiveness Outcomes/Quality metrics: Consistent with previous literature on the impact of hospital walking programs we will focus on hospital **length of stay** (LOS) due to its importance to patients and implications for overall facility efficiency and performance reporting (e.g., SAIL measures). Other important effectiveness outcomes will include inpatient fall rates (assessed using Inpatient Evaluation Center or IPEC), and discharge to nursing home (defined using established procedures combining VA + Medicare data sources).

Analyses. To address the question: *How should the STRIDE clinical program be adapted to optimize sustainability?* We will use the FRAME to classify STRIDE adaptations at 17 VAMCs that have started programs (via VISN 6 rollout, ongoing Function QUERI trial or other means). Data sources will include study team notes, communications, interview transcripts, and data collected on Diffusion Network calls. Responses will be coded and analyzed at site levels to develop facility-level summaries of STRIDE adaptations, indicating where there is divergence in responses at the site-level. From this coded data, we will identify and visually display emergent themes in a matrix, with columns reflecting implementation outcomes (i.e., fidelity and penetration) arranged from high to low, to illustrate patterns in program adaptations according to implementation outcomes. To address the question: *Are there differences in implementation outcomes (penetration, fidelity) at 10 months (primary), 13, 16 months between arms?* We hypothesize that sites randomized to enREP will have higher **penetration** (proportion of eligible patients with ≥ 1 STRIDE documented walk during hospitalization) and **fidelity** (eligible hospitalizations with at least one documented STRIDE walk as the percentage of eligible hospital days with "full dose" of the program, defined as two or more documented walks or one for more than five minutes.). We will use generalized linear models[75] to examine the effect of foundational vs. enREP on implementation outcomes at 10-months. The main predictors of interest will be foundational REP vs. enREP and stratification variables used in the covariate constrained randomization (described above). We will examine how fidelity and penetration outcomes change over time using descriptive methods (e.g. plots, descriptive statistics, subgroups). To address the question: *What is the impact on effectiveness outcomes and quality metrics (hospital length of stay or discharge to nursing home) at implementing sites?* In exploratory analyses, we are interested in assessing the impact of STRIDE on hospital LOS, discharge to nursing home, and inpatient fall rates. Our sample of Veterans for this analysis will

come from sites that adopt STRIDE (approximately 95,000 Veterans). The sample of Veterans will be from those who are hospitalized and meet our patient eligibility criteria; some of those patients will have participated in STRIDE (i.e. treatment group) and some will have not (i.e. comparison group). Data sources include CDW, VA/CMS repository data, GEC Health Care Risk measures and Geriatrics and Extended Care Data Analysis Center (GECDAC) Residential History File data. We will use inverse-probability of treatment weighted methods to adjust for confounding and estimate an average causal treatment effect for STRIDE. The study team will determine *a priori* which covariates are likely to be key determinants of receiving STRIDE. Qualitative data collection and approaches to data integration have been described previously.

Sample size estimate. Sample size calculations were conducted for the implementation outcomes at 10-months. Using a two-sided t-test based on a sample size of a minimum of 32 sites (at least 16 foundational REP and at least 16 enREP), a type-1 error rate of 5%, we will have 80% power to detect an effect size difference of 1.0 and 90% power to detect an effect size difference of 1.2 between arms. For the primary penetration implementation outcome, assuming a standard deviation of approximately 9% based on preliminary data from ongoing work, these effect size differences correspond to a mean difference of 9% between arms for 80% power and 10.8% for 90% power, with enREP having the higher penetrance.

Leadership. Dr. Hastings, a geriatrician, is the Corresponding PI for the Function QUERI program. Dr. Hastings leads Durham's COIN ADAPT, and has substantial clinical, research and leadership expertise relevant to implementing and evaluating new clinical programs in VA.

Implementation Project 3: Caregivers FIRST

Urgency of expanding supports to family caregivers and relationship to ICF. 2 million Veterans with functional and/or cognitive impairment in VA receive informal care, e.g., care from a family member or friend, and this support is a key determinant of whether or not function and independence are preserved. These caregivers are currently underserved; only 4000 are registered in VA's General Caregiver Support Program, and they report unmet needs.[85] Today, the policy environment supports substantially expanding services to general caregivers, e.g. those caring for Veterans from all service eras.

**Objective**	Improve caregiver and Veteran functioning through caregiver skill training and support
**Evidence**	Van Houtven et al. Family caregiver skills training to improve experiences of care: a randomized clinical trial. J Gen Intern Med 2019 Oct;34(10):2114-2122.
**Primary Partners**	VA Caregiver Support Program

Project Goal: The overall goal of the project is to implement, evaluate and sustain Caregivers FIRST in VAMCs throughout the country (maximum 150 sites). We will address the following specific aims:

<u>Specific Aim 1a</u>**:** Plan for and launch national implementation of Caregivers FIRST.

Key questions: What VACO and VISN partnerships and activities will enhance national dissemination of Caregivers FIRST? How should Caregivers FIRST clinical program be adapted to leverage site-specific resources and optimize sustainability?

<u>Specific Aim 1b</u>**:** Among sites that do not meet implementation adoption benchmarks, compare continuation of foundational REP versus addition of higher intensity strategies.

Key questions: Are there differences in implementation outcomes (penetration, fidelity) at 6, 12, or 18 months between arms? What is the impact on effectiveness outcomes/quality metrics (quality of VA General Caregiver Program, Veteran days in the community) at implementing sites? How do sites experience implementation strategies in each arm?

Specific Aim 2: Evaluate the effectiveness of Caregivers FIRST (exploratory).

Key questions: How does Caregivers FIRST impact key CSP Program of General Caregiver Support Services (PGCSS) quality metrics (inclusion of Caregivers FIRST in CSP site performance plans, number of caregivers enrolled in the CSP PGCSS)? How to the implementation strategies impact quality metrics? What is the effectiveness of Caregivers FIRST to increase Veteran patient's time at home over 6 months compared to similar patient whose caregivers did not participate in Caregivers FIRST?

Specific Aim 3: Conduct a business case analysis to identify cost-efficient strategies for facilitating the spread of Caregivers FIRST.

Key questions: What are the costs for delivering one round of Caregivers FIRST training? What are the costs of the high- and low-intensity implementation support?

Study design and methods

Aim 1 is addressed using an evidence-based sequence of activities to be ready to launch at national scale.[86] Specifically, the 'set-up' activities will be vital and include refining materials for foundational REP, revising CPRS/Cerner documentation templates, collaborate with the Caregiver Support Program in their building of a Caregivers FIRST data dashboard, and helping CSP prepare and field a brief national survey to assess site organizational readiness specific to Caregivers FIRST roll-out.[50] We will engage VAMCs with existing programs and other stakeholders about how to leverage site resources and suggested EBP adaptations national rollout. Consistent with the REP framework, we will retain the core (4 group classes) and modifiable (staffing model, optional phone/video training) components of Caregivers FIRST EBP.

Following announcement by the National Director of CSP of Caregivers FIRST national roll-out to all 150 VAMCs as a mandatory new program, all sites (n=150) will receive foundational REP for 6 months. In the CRT to address Aim 2 we will enroll up to 25 sites that did not meet adoption benchmarks (i.e. did not deliver a round of Caregivers FIRST in first 6-months or had low enrollment defined as 4 or less caregivers trained in the first 6-months). Based on the Roger's diffusion model,[87] we anticipate approximately 23% of sites will launch Caregivers FIRST within the first 6 months after national implementation is announced, with the remaining sites nonresponsive during this initial 6-month time period. A subset of the nonresponding sites (n=85) will be enrolled and randomized (up to n=25) to either continue foundational REP or receive higher intensity support strategies. To select the subset of sites to approach initially for enrollment and subsequent randomization, the non-responding sites will be prioritized by VISN, site complexity level, implementation activity (e.g., no implementation versus implementing but with low enrollment), and CSP staffing capacity (CSP total staffing FY20). The goal will be to select from nonresponding sites distributed across VISNs (approximately 2 per VISN if there are eligible sites) with sites systematically selected to approach for enrollment based on complexity level and lowest implementation activity within a VISN. In partnership with CSP, we will invite all CSP VISN Leads to encourage medical centers within their region to enroll. Once sites are enrolled a stratified, randomization will be conducted with site complexity level as 2 dichotomous stratification variables: 1) low complexity (2, 3 and no complexity) versus all others and 2) prior implementation of Caregivers FIRST (implementation in FY21 or FY22 vs. none). Additional site inclusion criteria include: (1) VISN lead and CSP champion willing to participate in study, willing to document class participation, and participate in assessments; (2) agree to start program within 6 months. We anticipate that the vast majority of non-adopting sites will agree to try to start a program because the announcement of Caregiver FIRST as a mandatory program provides strong incentives for them to be successful.

Implementation activities. Foundational REP activities have been described previously. After 6 months, sites that did not meet adoption benchmarks and were enrolled in the CRT (n=25) will either continue with foundational REP (n=12) or receive higher intensity implementation support (n=13). Continuing foundational REP for half of the sites is a reasonable approach because we cannot anticipate whether VAMCs that do not adopt Caregiver FIRST within the first 6 months

simply need a) more time given competing priorities or other reasons or b) a more intensive approach. Higher intensity implementation support over a period of approximately 4 months will consist of a needs assessment survey to assess barriers to implementation and approximately 3 to 4 facilitated group modules provided by Function QUERI and VA CSP to promote interactive problem solving in the context of a supportive interpersonal relationship.

Caregiver/Veteran participants. Targets for participation in Caregivers FIRST are family or friend caregivers of Veterans with functional and/or cognitive impairment, along with caregivers who have been identified as needing training, education and support (by Caregiver Support Program or others). The target class size for each round of the program is approximately 10-12 caregivers of Veterans, which would yield approximately 20-24 caregivers being trained per 6-month period if the recommended 2 rounds of classes are administered.

Outcomes. *Implementation outcomes*. **Adoption** is a cluster-level dichotomous outcome defined as meeting a threshold of four or more training classes delivered to a minimum of five caregivers over a cumulative 12-month period (yes) or not meeting this threshold is considered non-adoption (no). In addition, while 12 months is the primary endpoint, penetration and fidelity outcomes will also be assessed over 6- and 18-month time periods with descriptive results. **Penetration** (primary outcome) defined as the (1) proportion of caregivers who received consults for VA caregiver education and training services who attended at least one class at a medical center over a 12-month period and (2) the number of classes delivered over a 12-month period. **Fidelity** outcomes are cluster-level defined as (1) the proportion of the number of recommended classes delivered over a 12-month period, (2) the number of caregivers who attended at least one class over a 12-month period, and (3) the mean number of classes attended per caregiver over a 12-month period. *Effectiveness outcomes/ Quality metrics.* Effectiveness outcomes will include Veteran days in the community (e.g., not in a hospital or nursing home) over a 6 month period after caregiver participation in Caregivers FIRST. We will use the inclusion of Caregivers FIRST in VA medical center performance plans as the quality metric, where sites report the status on meeting requirements within the CSP Program of General Caregiver Support Services (PGCSS). In addition, we will evaluate the number of caregivers enrolled with each site's PGCSS and whether the quality metrics change based on the implementation approach.

Analyses. To address the question: *Are there differences in implementation outcomes (penetration, fidelity) at 6, 12, or 18 months between arms?* We hypothesize that sites randomized to higher intensity strategies will achieve higher **penetration** and **fidelity**. As part of our type III effectiveness-implementation hybrid design framework, the primary research question compares differences in implementation outcomes (penetration, fidelity, and adoption) between implementation arms at 12 months. Implementation outcomes are continuous, count, and binary outcomes, and generalized linear models[75] will be used to examine the effect of high-touch support on implementation outcomes at 12 months. The main predictor of interest will be enhanced (high touch) implementation support vs. foundational (low touch) implementation support, and stratification variables for site complexity (low complexity (2, 3, and no complexity) versus all others) and prior implementation of Caregivers FIRST or not will be included; other facility level covariates for consideration are CSP program quality score and size of CSP program. In secondary analyses, implementation outcomes over a 6-month and 18-month period will be assessed and described. We will examine how implementation outcomes change over time using descriptive methods (e.g. plots, descriptive statistics, subgroups). Descriptive statistics for survey measures such as organizational readiness for implementation change will be calculated overall and by randomization arm. We will use the same modeling approach described above to examine the effect of the implementation strategy on survey measures. To address the question: *What is the impact on effectiveness outcomes/quality metrics (quality of VA General Caregiver Program, Veteran days in the community) at implementing sites?* We will assess the impact of Caregivers FIRST on Veteran days in the

community (home time) over a 6-month period. Our sample of Veterans for this analysis will come from sites that adopt Caregivers FIRST nationally (that is, not limited to the 25 enrolled sites). The sample of Veterans (approximately 20,000) will be from those whose caregiver received consults for VA caregiver education and training services, including to Caregivers FIRST over a year period after the program was announced as a minimum standard; some of those caregivers will attend Caregivers FIRST classes (i.e., treatment group) and some will not (i.e., comparison group). Data sources include CDW, VA/CMS repository data, Caregiver Support Program Data, and Geriatrics and Extended Care Data Analysis Center (GECDAC) Residential History File data. We will use inverse-probability of treatment weighted methods to adjust for confounding and estimate an average causal treatment effect for Caregivers FIRST on home time over the 6-month time period following the consult for a VA caregiver education and training. We use the same modeling approach described above to examine the effect of implementation approach on general program quality scores and size of the general caregiver program. In exploratory analysis, we are interested in assessing the impact of Caregivers FIRST on Veteran's days in the community over a 6-month period. Our sample of Veterans for this analysis will come from sites that adopt Caregivers FIRST. The sample of Veterans will be from those whose caregiver received consults for VA caregiver education and training services, including to Caregivers FIRST over a year period; some of those caregivers will attend Caregivers FIRST classes (i.e. treatment group) and some will not (i.e. comparison group). We will use inverse-probability of treatment weighted methods to adjust for confounding and estimate an average causal treatment effect for Caregivers FIRST on home time over the 6-month time period following the consult for a VA caregiver education and training. The study team will determine *a priori* which covariates are likely to be key determinants of attending Caregivers FIRST training. We will explore the distribution that best fits the days at home outcome: Poisson, Negative Binomial, or zero-inflated. Generalized linear models with inverse propensity score weights will be used. We will describe the quality metrics using descriptive statistics, but these will not be modeled. Qualitative data collection and approaches to data integration have been described previously. *To address the key questions: What are the costs for delivering one round of Caregivers FIRST training? What are the costs of the high- and low-intensity implementation support?* The business case will include a budget impact analysis to help VA administrators consider the cost of implementation and delivery along with the effectiveness of the intervention in the context of the VA priorities to improve Veteran outcomes. The budget impact analysis will evaluate implementation costs for each strategy and intervention delivery costs per participant. The high-touch implementation support strategy is expected to use more resources (delivery time and attendance time) and potentially improve implementation outcomes, for example improved fidelity or increased penetration. To assess the value of the additional investment in implementation support, we will describe implementation costs per enrolled participant for each implementation strategy. While the overall resource use might be higher, a lower cost per participant would indicate a high value. We will use staff time and equipment data from the adaptations survey (baseline, 6 months, and 12 months) to assess costs using U.S. Veterans Health Administration salary and managerial cost accounting data. Drawing from methods in prior Function QUERI work, Business Case Analysis (BCA) will frame affordability to VA. For the cumulative 12-month study period, two types of costs will be collected for each site using a standardized method to track implementation activities 1) clinical delivery team costs incurred and 2) implementation strategy costs incurred. Because the 25 sites enrolled will represent a fraction of the sites nationally who will also implement (expect over 100 to implement), we can extrapolate to a national scenario in the BCA.

Sample size estimate. Sample size calculations were conducted for the penetration implementation outcomes at 12-months. Based on a two-sided t-test based on a sample size of 25 sites (12 foundational REP and 13 higher intensity), a type-1 error rate of 5%, we will have 80% power to detect an effect size difference of 1.2 and 90% power to detect an effect size

difference of 1.4 between arms. We did not adjust alpha for co-primary outcomes as success will be assessed based on improvement in both measurements of penetration **Leadership**. Dr. Courtney Van Houtven, health economist and health services researcher, evaluates delivery and costs of long-term services and supports, including interventions to support informal caregivers. She is the director of the QUERI Partnered Evaluation of the nationwide VA Caregiver Support Program (VA CARES) and the Function and Independence Scientific Core at the Durham COIN ADAPT.

Rapid Response Team

In addition to conducting EBP-specific implementation activities, Function QUERI will develop a Rapid Response team with the goal of completing up to 2 time-sensitive implementation or evaluation projects each year related to promoting function and independence and prevention of disability. The Rapid Response team will meet a need the Function QUERI team has encountered frequently in our work to date; partners request assistance with answering questions in near 'real-time' but all research resources are assigned to specific projects. The Rapid Response Team will apply elements of the CDC's "Data-to-Action Framework"[88] to ensure evaluation focus is feasible for a rapid analysis approach and meets program needs. In this team-based approach, program and evaluation staff connect early on and work together as partners to specify evaluation questions. In this way, both the evaluation and operations partners will have a shared understanding about the evaluation questions and approach and will produce actionable data for the partners. The team will use the following three steps to rapidly collect and analyze data, regardless of which method, qualitative or quantitative, is used: 1) Meet with the operations partner to clarify questions, needs, audience, and produce and deliver a memo from this discussion detailing these decisions. 2) Deliberate as a team to determine optimal approach for rapid data collection and analyses. 3) Present findings to operations partners via briefing or written report, depending on partner needs. Rapid Response Team leaders will work with program leadership and the Mentoring Core Director to assemble project teams consisting of Function QUERI investigators, staff and Function QUERI scholars. Every Rapid Response project will include a range of early career to senior clinicians and scientists. This structure enhances opportunities for professional development, provides fresh perspectives to inform project plans, and strengthens VA's implementation pipeline for the future. **Leadership**. Our Rapid Response Team leaders (Dr. Sperber: *qualitative/mixed methods*, Dr. Olsen: *quantitative/biostatistics*, Dr. Pavon: *clinical/geriatrics*) have the necessary methodologic expertise, clinical and partnered research experience to complete time-sensitive implementation projects that align with this QUERI's focus on supporting Veteran function and independence.

Mentoring Core

The overarching goal of the Function QUERI Mentoring Core will be **to** accelerate the adoption of evidence-based practices in VA by training the next generation of investigators focused on implementing and evaluating EBPs to maximize Veterans' function and independence. To meet this goal, the Mentoring Core will recruit outstanding, diverse fellows and junior faculty and provide them opportunities to enhance their knowledge, skills and experience in applying implementation science and working with operations partners and other stakeholders to improve VA health care.

Recruitment and Retention. The Mentoring Core will recruit 2 scholars per year for a 2-year training experience. We will recruit both clinician and non-clinician scientists. Trainees in fellowship programs sponsored by VA's Office of Academic Affiliations (OAA) and other early career VA researchers (e.g. Career Development Awardees) will be eligible to participate. We expect to have high interest in the mentoring program locally; however, for maximum reach, the

program will be non-residential such that trainees at any VA site are eligible to apply. In collaboration with OAA, QUERI, and other university and VA partners, we will advertise and invite applications for our first cohort to begin October 2021. We will recruit via unpaid advertisements, and networking and advertising at appropriate professional meetings (e.g., American Geriatric Society) and social media (e.g., Twitter). Already 7 potential fellows have expressed interest in the program.

Training Opportunity. The Durham VA and Duke University offer a rich environment for implementation science training. Over the last 35 years, Durham's COIN ADAPT has trained 105 clinicians and 38 non-clinician health services research fellows and 27 mentored HSR&D Career Development Awardees. In addition to the OAA health services research fellowship programs, ADAPT is a site for: (1) the National Clinician Scholars Program, a national leadership and research training program for MDs and nurses; and (2) VA Quality Scholars program, an interdisciplinary training program for clinicians focused on health care system improvement. In addition, the COIN ADAPT has had an Implementation Science Methods Lab since 2013. Duke's Department of Population Health Sciences is home to an NIH-funded K12 implementation science fellowship serving 6 scholars. These programs will provide us with a rich pool of candidates for our program and also offer opportunities to leverage existing resources and training experiences. For example, Function QUERI implementation scholars will participate in VA Quality Scholar webinars with topics related to quality improvement, methods and analysis and professional development. Scholars will work with their mentoring team and Core director to develop an individualized training plan. Available opportunities include: (1) didactic courses and webinars; (2) ongoing conferences; and (3) workshop programs.. All components can be attended in-person or remotely. Additionally, Function QUERI will support scholars to attend the annual AcademyHealth Dissemination and Implementation conference for supplemental training and networking opportunities.

Mentored Research Experience. During the 2-year period of training, scholars must acquire both the knowledge and practical skills to perform implementation research, accomplish a body of work significant enough to demonstrate competence as an investigator, and successfully compete for research funds to support implementation or evaluation projects. Scholars will have a primary mentor who is Function QUERI investigator. Supporting mentors can include both Function QUERI investigators and other researchers who have complementary areas of expertise that align with the scholars' training goals. At the start of the fellowship, the mentoring team will work with the scholar to plan a project to be completed within the 2-year period. This could include leadership of a secondary analysis related to one of the EBPs, co-leadership of a Rapid Response project, or leadership on a dissemination product related to implementation core methods or activities. Academic products are important for trainees and therefore we will set a metric that all trainees lead one manuscript during the training period. Additionally, there are many practical challenges and decision points in implementation work that are not common in less pragmatic research. Trainees will therefore participate in all meetings of one EBP or core, so they can experience and play a role in navigating these challenges. Furthermore, it is critical that trainees gain experience in working with operations and regional partners; scholars will participate in Technical Expert Panel (TEP) meetings for hands-on experience in stakeholder engagement and spend one week observing and learning about operations at VISN 6 headquarters, or their home VISN if non-residential. Combined, the program is carefully formulated to supplement scholars' existing and/or ongoing research training with the specialized skills and experience needed to implement and evaluate programs across VA.

Formal Evaluation. Responsible conduct of a training program requires careful monitoring of outcomes across stages and aspects of the program. Therefore, we will evaluate: 1)

Recruitment and selection of fellows, including applications and outcomes for underrepresented minorities and women; 2) Value of training activities from the perspectives of fellows and faculty; 3) The mentored research experience from the perspectives of scholars and mentoring faculty; 4) The record of presentations, peer-reviewed publications and other implementation products from each fellow; and 5) Post-program career experiences, including success in applying for and leading QUERI partnered evaluation projects and other grants, Diffusion of Excellence submissions, implementation science- or quality improvement-focused publications, and other implementation products such as playbooks.

Leadership. The Mentoring Core will be led by Dr. Hayden Bosworth, Deputy Director of COIN ADAPT and VA Senior Research Career Scientist. Dr. Bosworth is Vice Chair of Education for the Department of Population Health Sciences at Duke University and led ADAPT's OAA fellowship programs for 17 years. He currently leads the NIH K12 Dissemination and Implementation Science in Cardiovascular Outcomes program.

Stakeholder Alignment

Stakeholder engagement is a key underpinning of all Function QUERI activities to promote implementation and sustainment of EBPs. We will work closely with stakeholders through two primary mechanisms: Function QUERI TEP and Durham COIN ADAPT Veterans Research Engagement Panel (VetREP). The Function QUERI TEP includes key VA operations partners, VISN leaders and implementation science experts that align with our EBPs and aims. The TEP members include representatives from 5 different QUERIs, presenting a unique opportunity for cross-pollination of QUERI ideas and best practices. The TEP will convene annually, in person during years 1 and 3 and virtually in years 2 and 4. Meetings will involve discussion of VA national program office and VISN goals related to Function QUERI and how Function QUERI can optimally align with and contribute to those goals and clinical priorities, state of the art in implementation practice and science, review of milestones and presentation of key findings. We will also work closely with VetREP, a robust group that has been active for 3 years and has detailed procedures for member training, monthly meeting structure, and provision of feedback. The panel includes caregivers and Veterans with health conditions and experiences of high relevance to our EBPs. In year 1 we will solicit feedback from VetREP members on all 3 EBPs prior to launch. Thereafter in years 2-4, we will engage VetREP members at least twice annually in discussions relevant to ongoing Function QUERI activities, including dissemination of findings. We will communicate Function QUERI progress to all stakeholders through continued distribution of our quarterly newsletter.

Program Team and Management Plan

The Function QUERI team is comprised of an interdisciplinary group of investigators and clinicians with expertise in all aspects of the proposed program. Dr. Hastings, a geriatrician, is the Corresponding PI for the current Function QUERI program. In her roles as Director of the Durham COIN ADAPT, leader of VISN and GEC supported programs and HSR&D funded clinical trials, she has substantial expertise in leadership of implementation programs, local and national geriatric clinical practice, and partnered research. Dr. Hastings, Dr. Allen, and Dr. Van Houtven will lead Function QUERI's three EBP implementation projects. In addition to project directors, the leadership team is comprised of the Implementation Core (Zullig, Hughes), Rapid Response (Sperber, Olsen, Pavon) and Mentoring Core (Bosworth) Directors. Together, the team has the necessary expertise and experience required to successfully accomplish the aims of Function QUERI. Roles and tasks of each member of the investigative team and how the work will be coordinated are described in the Multiple PI plan. We propose a 4-year program of work for Function QUERI.

Risk/Benefit Assessment

There is no prospective patient or caregiver data collection proposed. All patient and caregiver data related to programs will be collected by clinical providers in the course of usual clinical care.

The key informant provider and stakeholder surveys and interviews are minimal risk and focus on organizational readiness, contextual factors and facilitators and barriers to implementation. They do not involve collection of quality of care or personal health information.

Additional Safeguards for Provider Interviews and Surveys. The information gained during the interviews and surveys will not be used to evaluate quality of care. Neither the provider's supervisors nor the provider's peers or patients will have access to the data. Data will remain confidential. No individually identifiable information will be published or disclosed, unless required by law.

In order to protect participants' privacy, we will avoid the use of names during the audiotaped interviews. To protect participants against risk after interview data have been collected, we will take a variety of measures to ensure confidentiality. In all records, participants will be assigned unique identifiers. The key linking unique identifiers to participants, the participation log, as well as all interview recordings and transcripts will be stored electronically in a restricted-use folder on a VA server. Any hard copies of these files will be stored in locked file cabinets in the offices of study personnel.

We will guard against the individual identification of participants in transcripts and research reports by using identifiers and by not identifying specific research sites. These data will be accessible only to the PIs, study staff and the appropriate data management personnel in Durham. The audio recordings to be transcribed will be placed in a dedicated sub-folder of the study's shared folder on the Durham HSR&D Project Drive, and approved Transcription staff, whether internal or external, will access files and complete tasks within this folder. No data will leave the Durham VAHCS. Electronic data will only be stored and accessed through secure VA servers and other VA information systems. Access to primary data will be restricted to those members of the research team with specific need to perform their duties. Data will not be removed from designated VA computer systems, except for archiving at study completion.

Protection of Data from Improper Use or Disclosure

There is a slight risk of breach of privacy resulting from unauthorized use, loss, or disclosures of PHI. However, if such an event occurs, the Durham local site PI and staff

will follow the Durham VA Health Care System's procedures. The lead principal investigator will work closely with the IRB, the ISO, and Privacy Officer if notification to individuals is necessary.

Through their VA training, all staff are familiar with the Durham VA Health Care System's procedure for reporting loss or theft of computer devices, unauthorized use, loss, or disclosures of PHI, or violations of information security requirements is to report the incident immediately (within 1 hour of discovery) to the VA Police, the employee's Supervisor, ISO, and Privacy Officer. Loss or theft of computer devices or PHI will also be reported to the Durham VA IRB as an adverse event.

<u>Benefits</u>. Veterans and caregivers may benefit from participation in the clinical programs (STRIDE, Group PT for Knee OA, Caregivers FIRST) that sites implement. Also, by participating in this project participants are contributing to knowledge that will help the VA better serve Veterans and caregivers in the future.

Selection of Subjects

There is no planned prospective enrollment of, or data collection from, patients or caregivers. Key informants (providers) will be surveyed/interviewed to inform the national implementation and evaluation of the evidence-based practices, deemed a quality improvement effort per certifying letters from national program office partners (see letters in Appendix). They will include members of the site implementation teams as well as organizational leaders (section chiefs, chief of staff, facility director). The selection of patients or caregivers for retrospective data collection in the EHR (implementation and effectiveness outcomes) are as follows below.

Group PT patient sample. Patients will be eligible for the Group PT if they have a clinician diagnosis of symptomatic knee OA. Participants will not be included if they have knee pain from another condition, have substantial fall risk, or have other co-occurring health conditions that would make participation in a group exercise class unsafe. EHR data collection will include program outcomes (adoption, penetration, fidelity) and effectiveness outcomes such as knee function and experience of care (p. 12).

STRIDE patient sample. Clinical criteria for STRIDE participation at each site will include: Age >= 60, admission for medical illness, community-dwelling (i.e. not in a nursing home), not requiring inpatient physical therapy, ability to follow one-step commands and to ambulate safely and independently. EHR data collection will include program outcomes (adoption, penetration, fidelity) and effectiveness outcomes such as length of stay and discharge to nursing home (p. 14).

Caregivers FIRST sample. Targets for participation in Caregivers FIRST are family or friend caregivers of Veterans with functional and/or cognitive impairment, along with

caregivers who have been identified as needing training, education and support (by Caregiver Support Program or others). The target class size for each round of the program is approximately 10-12 caregivers of Veterans, which would yield approximately 20-24 caregivers being trained per 6-month period if the recommended 2 rounds of classes are administered.   EHR data collection will include program outcomes (adoption, penetration, fidelity) and effectiveness outcomes such as Veteran days in the community (p. 16-17).

Subject Recruitment

There is no subject recruitment and enrollment for patients, caregivers or providers in this study. As noted above, key informants (providers) at each site will be surveyed and/or interviewed for QI purposes to inform implementation and evaluation of the clinical programs.

Consent Process

N/A

Study Interventions

N/A; observational study

Adverse Events

N/A

Costs and/or Payments to Subjects

N/A

Data and Safety Monitoring

This study carries minimal risk of unexpected or adverse events. The Durham HSR&D Center of Innovation to Accelerate Discovery and Practice Transformation (ADAPT) adheres to VA policy and Durham VAHCS IRB requirements, and also has developed additional Standard Operating Procedures (SOP) for safety monitoring and data security which have been designed to ensure continued confidentiality, integrity, and availability of research data. Privacy or information security incidents will be immediately reported.

With respect to all data, Durham ADAPT SOP procedures mandate the following to ensure confidentiality and safe handling of all data: (1) Access to all participant data and information will be restricted to authorized personnel; (2) Participants will not be identified by name in any reports or publications, nor will data be presented in such a way that the identity of individual participants can be inferred; (3) Each provider participant will be assigned an identifier which will be used on all study forms; and (4) All

study personnel will maintain certification with the Durham VAHCS IRB that they have completed training in research ethics and confidentiality.

With respect to paper-based records, these procedures mandate the following: (1) All study records that contain participant information will be kept in secured locked areas when not in use; and (2) In addition, such materials, when in use, will be kept safe from public scrutiny.

With respect to computer based records, the following practices are followed: (1) All research data are stored on VA-administered servers which are physically secured in a Durham VAHCS server room; (2) Individual computer accounts, password protected, are issued to staff members; and (3) Access to computer data is granted by OI&T personnel after confirming appropriate documentation through the IRB, per ADAPT policies. Utilization and other patient data will be downloaded directly from national files to the Durham HSR&D ADAPT servers or stored in the secure VINCI environment. Of study personnel, only the study Statisticians or other designated personnel will have access to the patient data, which will not be moved from this secured environment.

The key linking the identifiers to the providers' identifying information will be stored in a password protected electronic database and maintained on a password protected VA server, with access only available to approved study staff and investigators.

Withdrawal of Participants

N/A

Data Analysis and Statistical Considerations

The individual projects' sample size determination and analysis plans are as follows below. The data will be analyzed on SAS and SQL local servers (see Location of Data on p. 29) and in the secure VINCI environment by study statisticians and other relevant study team members listed on the IRB-approved study staff listing.

Group PT sample size estimate. Sample size calculations were conducted for the implementation outcomes at 12-months. Using a two-sided t-test based on a sample size of 16 sites (8 foundational REP and 8 enREP), a type-1 error rate of 5%, we will have 80% power to detect an effect size difference of 1.5 and 90% power to detect an effect size difference of 1.7 between arms. For the primary penetration outcome, assuming standard deviations ranging from 1.5 to 2.5 based on data of number of new patients per month over a 1-year period of implementation of Group PT in Durham, these effect size differences correspond to differences in mean number of initiators per month ranging from approximately 2.3 to 3.8 between arms for 80% power and 2.6 to 4.3 for 90% power.

Analyses. To address the question: *Are there differences in implementation outcomes (adoption, penetration, fidelity) at 6 and 12 months between arms?* We hypothesize that sites randomized to enREP will have superior *implementation outcomes*, including higher **adoption**, **penetration** and **fidelity**, compared with foundational REP sites at 12-months. We will use generalized linear models[75] to examine the effect of foundational vs. enREP on implementation outcomes at 12-months. The main predictors of interest will be Foundational REP vs. enREP and a stratification variable for the Complexity Level, where sites 1a, 1b, and 1c are one category and all others (2, 3, CBOCs/ Health Care Centers) are the other category and Rurality, where sites serving ≥50% rural/highly rural Veterans are one category and sites serving <50% rural/highly rural Veterans are another category. We will examine how implementation outcomes change over time using descriptive methods (e.g. plots, descriptive statistics, subgroups). To address the question: *What are effectiveness outcomes (function, pain) at implementing sites*? We will describe effectiveness/quality outcomes for patients who enroll in the Group PT program, overall and by study arm. We will calculate descriptive statistics for all visits, and change outcomes, among patients who have data from both the first and last visits. Qualitative data collection and approaches to data integration are described previously.

STRIDE sample size estimate. Sample size calculations were conducted for the implementation outcomes at 10-months. Using a two-sided t-test based on a sample size of 32 sites (16 foundational REP and 16 enREP), a type-1 error rate of 5%, we will have 80% power to detect an effect size difference of 1.0 and 90% power to detect an effect size difference of 1.2 between arms. For the primary penetration implementation outcome, assuming a standard deviation of approximately 9% based on preliminary data from ongoing work, these effect size differences correspond to a mean difference of 9% between arms for 80% power and 10.8% for 90% power, with enREP having the higher penetrance.

Analyses. To address the question: *How should the STRIDE clinical program be adapted to optimize sustainability?* We will use the FRAME to classify STRIDE adaptations at 17 VAMCs that have started programs (via VISN 6 rollout, ongoing Function QUERI trial or other means). Data sources will include study team notes, communications, interview transcripts, and data collected on Diffusion Network calls. Responses will be coded and analyzed at site levels to develop facility-level summaries of STRIDE adaptations, indicating where there is divergence in responses at the site-level. From this coded data, we will identify and visually display emergent themes in a matrix, with columns reflecting implementation outcomes (i.e., fidelity and penetration) arranged from high to low, to illustrate patterns in program adaptations according to implementation outcomes. To address the question: *Are there differences in implementation outcomes (penetration, fidelity) at 10 months (primary), 13, and 16 months between arms?* We hypothesize that sites randomized to enREP will have higher **penetration** (proportion of eligible patients with ≥ 1 STRIDE documented walk during hospitalization) and **fidelity** (higher proportion of STRIDE participants with ≥ one documented supervised walk, and with ≥ 10 minutes of daily walking). We will use generalized linear models[75] to examine the effect of foundational vs. enREP on implementation outcomes at 10-months. The main predictors of interest will be foundational REP vs. enREP and stratification variables used in the covariate constrained randomization (described above). We will examine how fidelity and penetration outcomes change over time using descriptive methods (e.g. plots, descriptive statistics,

subgroups). To address the question: *What is the impact on effectiveness outcomes and quality metrics (hospital length of stay or discharge to nursing home) at implementing sites?* In exploratory analyses, we are interested in assessing the impact of STRIDE on hospital LOS, discharge to nursing home, and inpatient fall rates. Our sample of Veterans for this analysis will come from sites that adopt STRIDE. The sample of Veterans will be from those who are hospitalized and meet our patient eligibility criteria; some of those patients will have participated in STRIDE (i.e. treatment group) and some will have not (i.e. comparison group). We will use inverse-probability of treatment weighted methods to adjust for confounding and estimate an average causal treatment effect for STRIDE. The study team will determine *a priori* which covariates are likely to be key determinants of receiving STRIDE. Qualitative data collection and approaches to data integration have been described previously.

Caregivers FIRST sample size estimate. Sample size calculations were conducted for the penetration implementation outcomes at 12-months. Based on a two-sided t-test based on a sample size of 25 sites (12 foundational REP and 13 higher intensity), a type-1 error rate of 5%, we will have 80% power to detect an effect size difference of 1.2 and 90% power to detect an effect size difference of 1.4 between arms. We did not adjust alpha for co-primary outcomes as success will be assessed based on improvement in both measurements of penetration. **Analyses.** To address the question: *Are there differences in implementation outcomes (penetration, fidelity) at 6, 12, or 18 months between arms?* We hypothesize that sites randomized to higher intensity strategies will achieve higher **penetration** and **fidelity**. As part of our type III effectiveness-implementation hybrid design framework, the primary research question compares differences in implementation outcomes (penetration, fidelity, and adoption) between implementation arms at 12 months. Implementation outcomes are continuous, count, and binary outcomes, and generalized linear models[75] will be used to examine the effect of high-touch support on implementation outcomes at 12 months. We will use generalized linear models[75] to examine the effect of implementation arm on implementation outcomes at 12-months. The main predictor of interest will be enhanced (high touch) implementation support vs. foundational (low touch) implementation support, and stratification variables for site complexity (low complexity (2, 3, and no complexity) versus all others) and prior implementation of Caregivers FIRST or not will be included; other facility level covariates for consideration are CSP program quality score and size of CSP program. In secondary analyses, implementation outcomes over a 6-month and 18-month period will be assessed and described.. We will examine how implementation outcomes change over time using descriptive methods (e.g. plots, descriptive statistics, subgroups). Descriptive statistics for survey measures such as organizational readiness for implementation change will be calculated overall and by randomization arm. We will use the same modeling approach described above to examine the effect of the implementation strategy on survey measures. To address the question: *What is the impact on effectiveness outcomes/quality metrics (quality of VA General Caregiver Program, Veteran days in the community) at implementing sites?* We will assess the impact of Caregivers FIRST on Veteran days in the community (home time) over a 6-month period. Our sample of Veterans for this analysis will come from sites that adopt Caregivers FIRST nationally (that is, not limited to the 25 enrolled sites). The sample of Veterans will be from those whose caregiver received consults for VA caregiver education and training services, including to Caregivers FIRST over a year period after the program was announced as a minimum standard; some of those caregivers will attend Caregivers FIRST classes (i.e., treatment group) and some will not (i.e., comparison group). We will use inverse-probability of treatment weighted methods to adjust for confounding and estimate an average causal treatment effect for Caregivers FIRST on home time over the 6-month time period following the consult for a VA caregiver education

and training. We use the same modeling approach described above to examine the effect of implementation approach on general program quality scores and size of the general caregiver program. In exploratory analysis, we are interested in assessing the impact of Caregivers FIRST on Veteran's days in the community over a 6-month period. Our sample of Veterans for this analysis will come from sites that adopt Caregivers FIRST. The sample of Veterans will be from those whose caregiver received consults for VA caregiver education and training services, including to Caregivers FIRST over a year period; some of those caregivers will attend Caregivers FIRST classes (i.e. treatment group) and some will not (i.e. comparison group). We will use inverse-probability of treatment weighted methods to adjust for confounding and estimate an average causal treatment effect for Caregivers FIRST on home time over the 6-month time period following the consult for a VA caregiver education and training. The study team will determine *a priori* which covariates are likely to be key determinants of attending Caregivers FIRST training. We will explore the distribution that best fits the days at home outcome: Poisson, Negative Binomial, or zero-inflated. Generalized linear models with inverse propensity score weights will be used. We will describe the quality metrics using descriptive statistics, but these will not be modeled. Qualitative data collection and approaches to data integration have been described previously.

Privacy, Confidentiality, and Information Security

1. Lists of Data Reviewed and/or Collected for Screening/Recruitment and Conduction of Study:

The Personal Health Information that will be obtained, used, and/or shared for this study includes:

Identifier(s)	Source(s) of Health Information
☒ Names	☒ Medical history & physical exam information
☒ All geographic subdivisions smaller than a State, including street address, city, county, precinct, and zip code. Describe: zip code needed for rurality variable	☐ Photographs, videotapes, audiotapes, or digital or other images
☒ All elements of dates (except year) for dates directly related to an individual, including birth date, admission date, discharge date, visit or treatment dates, etc.; and all ages over 89, Describe: birth date, admission and discharge dates, referral dates, ED visit dates, date of death, appointment date	☐ Biologic specimens (e.g., blood, tissue, urine, saliva). Describe:
☒ Telephone numbers	☒ Progress notes
☐ Fax numbers	☐ Diagnostic / Laboratory test results
☒ Electronic mail addresses - (VA emails)	☐ Operative reports
☒ Social Security Numbers	☐ Imaging (x-ray, CT, MRI, etc.)
☒ Medical record numbers	☒ Discharge summaries
☐ Health plan beneficiary numbers	☒ Survey / Questionnaire responses

Identifier(s)	Source(s) of Health Information
☐ Account numbers	☒ Billing records
☐ Certificate and/or license numbers	☐ HIV testing or infection records
☐ Vehicle identifiers and serial numbers, including license plate numbers	☐ Sickle cell anemia information
☐ Device identifiers and serial numbers	☐ Alcoholism or alcohol use information
☐ Web Universal Resource Locators (URLs)	☐ Drug abuse information
☐ Internet Protocol (IP) address numbers	☐ Mental health (not psychotherapy) notes
☐ Biometric identifiers, including finger & voice prints	☐ Psychological test results
☐ Full-face photographic images and any comparable images	☐ Genetic testing
☐ Any other unique identifying number, characteristic, or code, describe : **Note: This is not the unique code assigned to otherwise de-identified health information for re-identification purposes.*	☒ Other, describe: VA/CMS repository data, GECDAC Residential History File data , field and call notes including polls, site intake form.

2. Data and/or Specimen Acquisition:

Data for this study will be collected through (*check all that apply*):

☐ Prospective data and/or specimen collection obtained from participants. Provide description of processes: .

☒ Retrospective data collection and/or specimens obtained from medical chart review/data access.

Describe how data will be obtained (e.g., fileman, CDW, etc.): Program utilization and outcomes data will be downloaded directly from national files to the Durham HSR&D ADAPT servers or the secure VINCI environment. VA/CMS Repository data and GECDAC Residential History File data will be obtained from VIREC after approval of a DUA. Health factor data and other EHR may also be reviewed through JLV.

☒ Retrospective data collection and/or specimens obtained from an IRB-approved data and/or specimen repository. Indicate the repository source including name, VA location, and IRB number: VA/CMS Repository data, and GECDAC Residential History File data .

Note: for data and/or specimens obtained from a VA approved data repository, a Data Use Agreement (DUA) must be executed prior to obtaining data and/or specimens. See VHA Handbook 1200.12 for further information.

3. Level of Data:

The following level(s) of data will be acquired/maintained for this study (*check all that apply*):

☒ Identifiable—Data contains direct identifiers.
☐ Coded—Data linked to a specific by a code rather than a direct identifier for re-identification purposes. Only someone possessing the key to the code can link the data to a particular participant.
☐ De-Identified (all 18 HIPAA identifiers removed
  ☐ Verified Statistically
    OR
  ☐ Verified by Absence or Removal of 18 HIPAA identifiers
☐ Limited Data Set
☐ Other: Describe:

4. Location of Data and/or Specimens, and Data Retention Plan:

A. Data and/or Specimen Location:

Data will be stored electronically in \\OITDURHSMSMB601.va.gov\Durham_HSRD_R (in a restricted-access study folder), \\Vhadurappsas1.v06.med.va.gov (SAS server temporary storage), and VA BOX account (https://veteransaffairs.ent.box.com/). Data that will be stored electronically include the data listed in "Selection of Subjects" and "Data Analysis and Statistical Considerations" above. Data stored on BOX is documented in VA Data Security Categorization Memorandum of Understanding (eMASS ID #2045) and will not exceed the "moderate" System Security Categorization.

No paper records of data are anticipated. If any paper records of data need to be generated, they will be stored in locked file cabinets in staff offices in HSR&D ADAPT.

☒ Data will be also be placed at the VA Informatics and Computing Interface (VINCI; http://vaww.vinci.med.va.gov/vincicentral/VINCIWorkspace.aspx). The VA Informatics and Computing Infrastructure is a partnership between the VA Office of Information Technology and the Veterans' Health Administration Office of Research and Development. Researchers and operations staff can use VINCI to access data and statistical analysis tools in a virtual working environment through a certified VHA network computer using the VA Intranet or Virtual Private Network (VPN).

B. Data Retention Plan

☒ Research records will be maintained and destroyed according to the National Archives and Records Administration, Records Schedule Number: DAA-0015-2015-0004. Records destruction, when authorized, will be accomplished using the then current requirements for the secure disposal of paper and electronic records. Currently, destruction of research records (see DAA-0015-2015-0004, section 7.6 "Research

Investigator Files” for materials included in research records) is scheduled for 6 years after the cut-off (the cut-off is the completion of the research project) and may be retained longer if required by other federal agencies. Records will not be destroyed without pre-notification to the facility records manager.

☐ Other data retention plan, describe:

5. Data Access and Data Recipients:

Only members of our Durham VA Health Care System research team (on our IRB-approved staff listing) will have access to data with identifiers, either behind the VA firewall or in the VINCI secure environment, for analysis of data.

All VA research personnel who have access to VHA records are instructed, in accordance with VA policy, on the requirements of Federal privacy and information laws and regulations, VA regulations and policies, and VHA policy. All study personnel who are VA employees working within the VA system have fulfilled all required HIPAA and other VA security and privacy policy training requirements and have agreed to follow guidelines pertaining to the protection of patient data. All research staff sign VA Rules of Behavior, and all study staff are up-to-date with VHA Privacy Policy Training and the VA Office of Cyber and Information Security Awareness Training Course. The data security and privacy procedures summarized in that course include logging off or locking the computer when walking away from it; no sharing of access codes, verify codes or passwords; not allowing anyone else to use the computer under one’s password; and disposing of sensitive information using VA-approved methods (e.g., shredder bins).

Access to study data will be removed for all study personnel when they are no longer part of the research team.

6. Data and/or Specimen Transportation and/or Transmission for all data and/or specimens involved in the study:

I. ☐ Data and/or specimens will <u>not</u> be transported or transmitted outside of Durham VAMC environment.

II. ☐ Data and/or specimens will be transported BETWEEN sites that are under the auspices of the Durham VA Medical Center.

III. ☐ Data and/or specimens will be transmitted to <u>other VA sites</u> using the following method(s):

A. Data

☐ Data are de-identified and thus will be sent via unencrypted e-mail or unencrypted disk (encryption is optional).

☐ Data are coded or contain identifiers and thus will be sent ☐ Other, describe:

B. Specimens

☐ Specimens are de-identified and thus will be sent via standard carrier (tracking is optional).

☐ Specimens are coded or contain identifiers and thus will be sent via VA-authorized carrier with tracking.

☐ Other, describe:

IV. ☒ Data and/or specimens will be transported to <u>non-VA/VHA sites (e.g., academic affiliates, laboratories, etc.)</u> using the following method(s):

A. Data

☐ Data are de-identified and thus will be sent via unencrypted e-mail or unencrypted CD.

☐ Data are coded or contain identifiers and thus will be sent via using VA—approved carrier with tracking.

☐ Data are coded or identified and will be sent via the Safe Access File Exchange (SAFE) at https://safe.amrdec.army.mil/safe/. SAFE is a secure method of exchanging files <2GB to and from individuals with a valid .gov, .mil, .com, or .edu email address. <insert information including collaborator name.>

☐ Data are coded or identified and will be uploaded to sponsor website using electronic case report form (eCRF)

☒ Other, describe: The VA's Qualtrics Research will be used to collect data

B. Specimens

☐ Specimens are de-identified and thus will be sent via standard carrier (tracking is optional) or will be hand-delivered by research study personnel. Specify method of delivery:

☐ Specimens are coded and thus will be sent via VA-approved carrier with tracking or will be hand-delivered by research study personnel. Specify method of delivery:

In accordance with the HIPAA and the Privacy Act, for any coded or identifiable data or specimens released from the Durham VAMC (with the exception of Limited Data Sets), an Accounting of Disclosure (AOD) will be maintained (e.g., in a database or spreadsheet) that includes the participant's name, date of the disclosure, description of

the nature of the Individually Identifiable Information (III) disclosed, purpose of each disclosure, and the name and address of the person/agency to whom the disclosure was made.

C. ☐ Local DVAMC memorandum “Authorization to Use, Process, Store, or Transmit VA Sensitive Information Outside VA Owned or Managed Facilities” has been pre-filled out for each study team member who may transport the data and/or specimens off-site. This (these) forms are included with the IRB materials.

D. ☐ Containers (e.g., briefcase, bin) are labeled with the following notice (label placed on the outside of container) in accordance with VHA Directive 6609:

NOTICE!!!

Access to these records is limited to: AUTHORIZED PERSONS ONLY. Information may not be disclosed from this file unless permitted by all applicable legal authorities, which may include the Privacy Act; 38 U.S.C. §§ 5701, 5705, 7332; the Health Insurance Portability and Accountability Act; and regulations implementing those provisions, at 38 C.F.R. §§ 1.460 – 1.599 and 45 C.F.R. Parts 160 and 164. Anyone who discloses information in violation of the above provisions may subject to civil and criminal penalties.

V. ☐ We will communicate with veterans enrolled as participants in this research study through MyHealtheVet.

7. Risk Mitigation Strategies:

All study staff are current on their privacy and security training and work to ensure the privacy and security of the data. Study policy is to provide locations to sensitive information using file paths in secure email rather than attaching documents. In addition to the PI, the study coordinator, statisticians and computer programmers provide an additional level of monitoring for data security.

☒ Data are fully de-identified (stripped of HIPAA 18 and study ID/code) before being shared outside of Durham VAMC. (NOTE: study has no plans to share data outside the Durham VAMC)

☐ Specimens are fully de-identified (stripped of HIPAA 18 and study ID/code before being shared outside of Durham VAMC.

☐ Data or specimens are coded and the code is not related to, or derived from, information about the individual and that code is not otherwise capable of being translated as to the identify the individual. Only someone possessing the key to code can link the data to a particular participant.

☐ Other, specify:

8. Suspected Loss of VA Information:

Should any incident such as theft or loss of data, unauthorized access of sensitive data or non-compliance with security controls occur it will be immediately reported according to VA policy. All incidents regarding information security/privacy incidents will be reported to the ISO and PO within 1 hour of acknowledgement of issue and done so using the VHADUR Research Events Report e-mail group (VHADURResearchEventReport@va.gov).

9. Reporting of Results:

☒ Reporting of results, such as in scientific papers and presentations, will never identify individual subjects. Data will be presented in aggregate and individual-level data will not be published.

☐ Other results reporting plan, describe:

10. Future Use of Data:

☐ Data will be retained for future use. This is described elsewhere in the protocol and is noted in the HIPAA authorization.

- ☐ Future Use of data is optional (i.e., not required by the research subject).
- ☐ Future Use of data is required for participation in the study.

☒ No future use of data is currently planned.

11. Use of Mail Merge Technology

☐ Mail merge programs will be used to generate letters and/or address labels for mailings to potential or already enrolled research subjects. The study team is aware that to reduce risk of mail merge related privacy incidents, use of mail merge programs requires a 25% accuracy check to verify that (potential) research subject name and mailing address are properly “matched”. If discrepancies are found, a 100% accuracy check is required before letters may be mailed.

12. Use of Non-Standard Software

☒ I do NOT intend to use any new specialized software (i.e. Software that’s not already approved OR installed) in this study.

☐ I intend to use specialized software that has not already been installed and it has been approved for use by the VA Technical Reference Model (TRM) Group. (Note: All new software must be approved by TRM before it can be installed on VA systems.)

☒ I intend to use previously installed software on my VA computer.

Previously approved versions of Atlas.ti and NVivo software will be used for qualitative data analysis.

13. Use of Cloud Computing Services

☒ Cloud computing services will NOT be used in this study.

☐ Cloud computing services WILL be used in this study as described below and have been approved nationally by the VA Chief Information Officer (CIO). (Note: ONLY cloud computing services that have been approved nationally may be used.)

References

1. Gill TM, Allore HG, Gahbauer EA, Murphy TE. Change in disability after hospitalization or restricted activity in older persons. *JAMA.* 2010;304(17):1919-1928.
2. Goulet JL, Kerns RD, Bair M, et al. The musculoskeletal diagnosis cohort: examining pain and pain care among veterans. *Pain.* 2016;157(8):1696-1703.
3. Meneses SR, Goode AP, Nelson AE, et al. Clinical algorithms to aid osteoarthritis guideline dissemination. *Osteoarthritis Cartilage*. 2016;24(9):1487-1499.
4. Abbate LM, Jeffreys AS, Coffman CJ, et al. Demographic and clinical factors associated with nonsurgical osteoarthritis treatment among patients in outpatient clinics. *Arthritis Care Res.* 2018;70(8):1141-1149.
5. Allen KD, Bongiorni D, Bosworth HB, et al. Group versus individual physical therapy for veterans with knee osteoarthritis: randomized clinical trial. *Phys Ther.* 2016;96(5):597-608.
6. Covinsky KE, Pierluissi E, Johnston CB. Hospitalization-associated disability. *JAMA.* 2011;306(16):1782-1794.
7. Hastings SN, Sloane R, Morey MC, Pavon JM, Hoenig H. Assisted early mobility for hospitalized older veterans: preliminary data from the STRIDE program. *J Am Geriatr Soc.* 2014;62(11):2180-2184.
8. Brown CJ, Foley KT, Lowman JD, Jr., et al. Comparison of posthospitalization function and community mobility in hospital mobility program and usual care patients: a randomized clinical trial. *JAMA Intern Med.* 2016;176(7):921-927.
9. Fisher SR, Kuo YF, Graham JE, Ottenbacher KJ, Ostir GV. Early ambulation and length of stay in older adults hospitalized for acute illness. *Arch Intern Med*. 2010;170(21):1942-1943.
10. Growdon ME, Shorr RI, Inouye SK. The tension between promoting mobility and preventing falls in the hospital. *JAMA Intern Med.* 2017;177(6):759-760.
11. Kalisch BJ, Lee S, Dabney BW. Outcomes of inpatient mobilization: a literature review. *J Clin Nurs.* 2014;23(11-12):1486-1501.
12. Liu B, Moore JE, Almaawiy U, et al. Outcomes of mobilisation of vulnerable elders in Ontario (MOVE ON): a multisite interrupted time series evaluation of an implementation intervention to increase patient mobilisation. *Age Ageing.* 2018;47(1):112-119.
13. Ostir GV, Berges IM, Kuo YF, Goodwin JS, Fisher SR, Guralnik JM. Mobility activity and its value as a prognostic indicator of survival in hospitalized older adults. *J Am Geriatr Soc.* 2013;61(4):551-557.
14. Pashikanti L, Von Ah D. Impact of early mobilization protocol on the medical-surgical inpatient population: an integrated review of literature. *Clin Nurse Spec.* 2012;26(2):87-94.
15. Mundy LM. Early mobilization of patients hospitalized with community-acquired pneumonia. *Chest.* 2003;124(3):883-889.
16. Sperber NR, Bruening RA, Choate A, et al. Implementing a mandated program across a regional health care system: a rapid qualitative assessment to evaluate early implementation strategies. *Qual Manag Health Care.* 2019;28(3):147-154.
17. Hastings SN, Choate AL, Mahanna EP, et al. Early mobility in the hospital: lessons learned from the STRIDE Program. *Geriatrics.* 2018;3(4).
18. Van Houtven CH, Smith VA, Lindquist JH, et al. Family caregiver skills training to improve experiences of care: a randomized clinical trial. *J Gen Intern Med.* 2019;34(10):2114-2122.
19. Lobb R, Colditz GA. Implementation science and its application to population health. *Annu Rev Public Health.* 2013;34:235-251.
20. Shepherd-Banigan M, Kaufman BG, Decosimo K, et al. Adaptation and Implementation

of a family caregiver skills training program: from single site RCT to multisite pragmatic intervention. [published online ahead of print September 9, 2019]. *J Nurs Scholarsh.* doi:10.1111/jnu.12511.

21. Wang V, Allen K, Van Houtven CH, et al. Supporting teams to optimize function and independence in Veterans: a multi-study program and mixed methods protocol. *Implement Sci.* 2018;13(1):58.
22. Van Houtven CH, Hastings SN, Colon-Emeric C. A path to high-quality team-based care for people with serious illness. *Health Aff.* 2019;38(6).
23. Cary MP, Goode V, Crego N, et al. Hospital readmission and costs of total knee replacement surgery in 2009 and 2014: potential implications for health care managers. *Health Care Manag.* 2019;38(1):24-28.
24. Wolff JL, Drabo EF, Van Houtven CH. Beyond parental leave: paid family leave for an aging America. *J Am Geriatr Soc.* 2019;67(7):1322-1324.
25. Van Houtven CH. Bringing invisible partners in care out of the shadows: employment effects of informal care provision in Europe and implications for the United States. *Health Serv Res.* 2018;53(4):2011-2019.
26. Van Houtven CH. Integrating health care and social services for people with serious illness: proceedings of a workshop. Paper presented at: National Academies of Science, Engineering, and Medicine; January 10, 2019; Washington, DC.
27. Kilbourne AM, Goodrich DE, Miake-Lye I, Braganza MZ, Bowersox NW. Quality enhancement research initiative implementation roadmap: toward sustainability of evidence-based practices in a learning health system. *Med Care.* 2019;57(suppl 10 suppl 3):S286-S293.
28. Chambers DA, Glasgow RE, Stange KC. The dynamic sustainability framework: addressing the paradox of sustainment amid ongoing change. *Implement Sci.* 2013;8:117.
29. Pype P, Mertens F, Helewaut F, Krystallidou D. Healthcare teams as complex adaptive systems: understanding team behaviour through team members' perception of interpersonal interaction. *BMC Health Serv Res.* 2018;18(1):570.
30. Proctor E, Silmere H, Raghavan R, et al. Outcomes for implementation research: conceptual distinctions, measurement challenges, and research agenda. *Adm Policy Ment Health.* 2011;38(2):65-76.
31. Kilbourne AM, Neumann MS, Pincus HA, Bauer MS, Stall R. Implementing evidence-based interventions in health care: application of the replicating effective programs framework. *Implement Sci.* 2007;2:42.
32. Kilbourne AM, Abraham KM, Goodrich DE, et al. Cluster randomized adaptive implementation trial comparing a standard versus enhanced implementation intervention to improve uptake of an effective re-engagement program for patients with serious mental illness. *Implement Sci.* 2013;8:136.
33. Bauer MS, Damschroder L, Hagedorn H, Smith J, Kilbourne AM. An introduction to implementation science for the non-specialist. *BMC Psychol.* 2015;3:32.
34. Wiltsey Stirman S, Baumann AA, Miller CJ. The FRAME: an expanded framework for reporting adaptations and modifications to evidence-based interventions. *Implement Sci.* 2019;14(1):58.
35. Richardson JL, Milam J, McCutchan A, et al. Effect of brief safer-sex counseling by medical providers to HIV-1 seropositive patients: a multi-clinic assessment. *Aids.* 2004;18(8):1179-1186.
36. Kelly JA, Somlai AM, DiFranceisco WJ, et al. Bridging the gap between the science and service of HIV prevention: transferring effective research-based HIV prevention interventions to community AIDS service providers. *American journal of public health.* 2000;90(7):1082-1088.

37. Powell BJ, Waltz TJ, Chinman MJ, et al. A refined compilation of implementation strategies: results from the Expert Recommendations for Implementing Change (ERIC) project. *Implement Sci.* 2015;10:21.
38. Colon-Emeric CS, McConnell E, Pinheiro SO, et al. CONNECT for better fall prevention in nursing homes: results from a pilot intervention study. *J Am Geriatr Soc.* 2013;61(12):2150-2159.
39. Colon-Emeric CS, Corazzini K, McConnell ES, et al. Effect of Promoting High-Quality Staff Interactions on Fall Prevention in Nursing Homes: A Cluster-Randomized Trial. *JAMA Intern Med.* 2017.
40. Colon-Emeric CS, Pinheiro SO, Anderson RA, et al. Connecting the learners: improving uptake of a nursing home educational program by focusing on staff interactions. *Gerontologist.* 2014;54(3):446-459.
41. Creswell JW CV. *Designing and conducting mixed methods research.* Sage Publications; 2017.
42. Proctor EK, Powell BJ, McMillen JC. Implementation strategies: recommendations for specifying and reporting. *Implement Sci.* 2013;8:139.
43. Hsieh HF, Shannon SE. Three approaches to qualitative content analysis. *Qual Health Res.* 2005;15(9):1277-1288.
44. Waltz TJ, Powell BJ, Matthieu MM, et al. Use of concept mapping to characterize relationships among implementation strategies and assess their feasibility and importance: results from the Expert Recommendations for Implementing Change (ERIC) study. *Implement Sci.* 2015;10:109.
45. Gale NK, Heath G, Cameron E, Rashid S, Redwood S. Using the framework method for the analysis of qualitative data in multi-disciplinary health research. *BMC medical research methodology.* 2013;13:117.
46. Morse JM, Niehaus L. *Mixed Method Design: Principles and Procedures.* Walnut Creek, CA: Left Coast Press; 2009.
47. Shortell SM, Zimmerman JE, Rousseau DM, et al. The performance of intensive care units: does good management make a difference? *Med Care.* 1994;32(5):508-525.
48. Anderson RA, Plowman D, Corazzini K, et al. Participation in decision making as a property of complex adaptive systems: developing and testing a measure. *Nurs Res Pract.* 2013;2013:706842.
49. Lee A, Vargo, J., Seville, E. Developing a tool to measure and compare organizations' resilience. *Nat Hazards Rev.* 2013;14:29-41.
50. Helfrich CD, Li YF, Sharp ND, Sales AE. Organizational readiness to change assessment (ORCA): development of an instrument based on the Promoting Action on Research in Health Services (PARIHS) framework. *Implement Sci.* 2009;4:38.
51. Vogus TJ, Sutcliffe KM. The Safety Organizing Scale: development and validation of a behavioral measure of safety culture in hospital nursing units. *Med Care.* 2007;45(1):46-54.
52. Mallak LA, Yildiz M. Developing a workplace resilience instrument. *Work.* 2016;54(2):241-253.
53. Weiner BJ, Amick H, Lee SY. Conceptualization and measurement of organizational readiness for change: a review of the literature in health services research and other fields. *Med Care Res Rev.* 2008;65(4):379-436.
54. Sullivan SD, Mauskopf JA, Augustovski F, et al. Budget impact analysis-principles of good practice: report of the ISPOR 2012 Budget Impact Analysis Good Practice II Task Force. *Value Health.* 2014;17(1):5-14.
55. Liu CF, Rubenstein LV, Kirchner JE, et al. Organizational cost of quality improvement for depression care. *Health Serv Res.* 2009;44(1):225-244.
56. Leatherman S, Berwick D, Iles D, et al. The business case for quality: case studies and

an analysis. *Health Aff.* 2003;22(2):17-30.

57. Curran GM, Bauer M, Mittman B, Pyne JM, Stetler C. Effectiveness-implementation hybrid designs: combining elements of clinical effectiveness and implementation research to enhance public health impact. *Med Care.* 2012;50(3):217-226.
58. Deshpande BR, Katz JN, Solomon DH, et al. Number of persons with symptomatic knee osteoarthritis in the US: impact of race and ethnicity, age, sex, and obesity. *Arthritis Care Res.* 2016;68(12):1743-1750.
59. Murphy LB, Helmick CG, Allen KD, et al. Arthritis among veterans - United States, 2011-2013. *MMWR Morbidity and Mortality Weekly Report.* 2014;63(44):999-1003.
60. Rivera JC, Wenke JC, Buckwalter JA, Ficke JR, Johnson AE. Posttraumatic osteoarthritis caused by battlefield injuries: the primary source of disability in warriors. *J Am Acad Orthop Surg.* 2012;20(suppl 1):S64-69.
61. Cameron KL, Hsiao MS, Owens BD, Burks R, Svoboda SJ. Incidence of physician-diagnosed osteoarthritis among active duty United States military service members. *Arthritis Rheum.* 2011;63(10):2974-2982.
62. Dominick KL, Golightly YM, Jackson GL. Arthritis prevalence and symptoms among U.S. non-veterans, veterans, and veterans receiving Department of Veterans Affairs health care. *J Rheumatol.* 2006;33:348-354.
63. Centers for Disease Control and Prevention. Projected state-specific increases in self-reported doctor-diagnosed arthritis and arthritis-attributable activity limitations--United States, 2005-2030. *MMWR Morbidity and Mortality Weekly Report.* 2007;56(17):423-425.
64. Cross M, Smith E, Hoy D, et al. The global burden of hip and knee osteoarthritis: estimates from the global burden of disease 2010 study. *Ann Rheum Dis.* 2014;73(7):1323-1330.
65. Menon J, Mishra P. Health care resource use, health care expenditures and absenteeism costs associated with osteoarthritis in US healthcare system. *Osteoarthritis Cartilage.* 2018;26(4):480-484.
66. Guglielmo D, Hootman JM, Boring MA, et al. Symptoms of anxiety and depression among adults with arthritis - United States, 2015-2017. *MMWR Morbidity and Mortality Weekly Report.* 2018;67(39):1081-1087.
67. Taylor SS, Hughes JM, Coffman CJ, et al. Prevalence of and characteristics associated with insomnia and obstructive sleep apnea among veterans with knee and hip osteoarthritis. *BMC Musculoskelet Disord.* 2018;19(1):79.
68. Dore AL, Golightly YM, Mercer VS, et al. Lower-extremity osteoarthritis and the risk of falls in a community-based longitudinal study of adults with and without osteoarthritis. *Arthritis Care Res.* 2015;67(5):633-639.
69. Corsi M, Alvarez C, Callahan LF, et al. Contributions of symptomatic osteoarthritis and physical function to incident cardiovascular disease. *BMC Musculoskelet Disord.* 2018;19(1):393.
70. Hawker GA, Croxford R, Bierman AS, et al. All-cause mortality and serious cardiovascular events in people with hip and knee osteoarthritis: a population based cohort study. *PLoS One.* 2014;9(3):e91286.
71. Health VOoR. Fact sheet: Information about the VHA Office of Rural Health and Rural Veterans, 2014. https://www.ruralhealth.va.gov/docs/factsheets/ORH_General_FactSheet_2014.pdf. Accessed December 9, 2019.
72. Bellamy N. WOMAC: a 20-year experiential review of a patient-centered self-reported health status questionnaire. *J Rheumatol.* 2002;29(12):2473-2476.
73. Bellamy N. *WOMAC Osteoarthritis Index User Guide. Version V.* Brisbane, Australia; 2002.

74. Bellamy N, Buchanan WW, Goldsmith CH, Campbell J, Stitt LW. Validation study of WOMAC: a health status instrument for measuring clinically important patient relevant outcomes to antirheumatic drug therapy in patients with osteoarthritis of the hip or knee. *J Rheumatol.* 1988;15:1833-1840.
75. McCullagh P, Nelder, John. *Generalized Linear Models.* Second Edition ed. Boca Raton, FL: Chapman and Hall/CRC; 1989.
76. Zisberg A, Shadmi E, Sinoff G, Gur-Yaish N, Srulovici E, Admi H. Low mobility during hospitalization and functional decline in older adults. *J Am Geriatr Soc.* 2011;59(2):266-273.
77. Brown CJ, Redden DT, Flood KL, Allman RM. The underrecognized epidemic of low mobility during hospitalization of older adults. *J Am Geriatr Soc.* 2009;57(9):1660-1665.
78. Murphy EA. A Key Step for Hospitalized Elders. *Arch Intern Med.* 2011;171(3):268-269.
79. Bailey M. Overzealous in preventing falls, hospitals are producing an ‘epidemic of immobility’ in elderly patients. *Washington Post.* October 13, 2019;Health.
80. Turner EL, Prague M, Gallis JA, Li F, Murray DM. Review of recent methodological developments in group-randomized trials: part 2-analysis. *Am J Public Health.* 2017;107(7):1078-1086.
81. Raab GM, Butcher I. Balance in cluster randomized trials. *Stat Med.* 2001;20(3):351-365.
82. Li F, Turner EL, Heagerty PJ, Murray DM, Vollmer WM, DeLong ER. An evaluation of constrained randomization for the design and analysis of group-randomized trials with binary outcomes. *Stat Med.* 2017;36(24):3791-3806.
83. Carter BR, Hood K. Balance algorithm for cluster randomized trials. *BMC Med Res Methodol.* 2008;8:65.
84. Miake-Lye I, Mak SS, Lambert-Kerzner AC, et al. *Scaling Beyond Early Adopters: A Systematic Review and Key Informant Perspectives*. Washington, DC: Department of Veterans Affairs; 2019.
85. Van Houtven CH, Oddone EZ, Weinberger M. Informal and formal care infrastructure and perceived need for caregiver training for frail US veterans referred to home and community-based services. *Chronic Illn.* 2010;6(1):57-66.
86. Barker PM, Reid A, Schall MW. A framework for scaling up health interventions: lessons from large-scale improvement initiatives in Africa. *Implement Sci.* 2016;11:12.
87. Rogers EM. *Diffusion of Innovations.* 4th ed. New York, NY: Simon & Schuster; 2010.
88. Zakocs R, Hill JA, Brown P, Wheaton J, Freire KE. The data-to-action framework: a rapid program improvement process. *Health Educ Behav.* 2015;42(4):471-479.

Appendix A: Expedited Review of Research

The categories of research that may be reviewed by the IRB through the expedited review procedure include research activities that present no more than minimal risk to human subjects **AND** involve procedures listed in one or more of the specific categories listed below.

The expedited review procedure is <u>not</u> to be used when identification of the subjects or their responses would reasonably place them at risk of criminal or civil liability; be damaging to the subjects' financial standing, employability, insurability, or reputation; or be stigmatizing, unless reasonable and appropriate protections are implemented so that risks related to invasion of privacy and breach of confidentiality are no greater than minimal. The IRB must apply the standard requirements for informed consent (or its waiver, alteration, or exception) to all studies that undergo expedited review.

EXPEDITE CATEGORIES
**1-Drugs and Devices:** One of the following must be met:<br>(1) The research is on drugs for which an IND application is not required.<br>(2) The research is on medical devices for which an investigational device exemption (IDE) application is not required; or the medical device is cleared or approved for marketing, and the medical device is being used in accordance with its cleared or approved labeling.
**2-Blood Samples:** Collected by finger / heel / ear stick or venipuncture:<br>(1) From healthy, nonpregnant adults who weigh at least 110 pounds. For these subjects, the amounts drawn may not exceed 550 milliliters (ml) in an 8-week period, and collection may not occur more frequently than two times per week; or<br>(2) From other adults and children, considering the age, weight, and health of the subjects, the collection procedure, the amount of blood to be collected, and the frequency with which it will be collected. For these subjects, the amount drawn may not exceed the lesser of 50 ml or 3 ml per kilogram (kg) in an 8-week period, and collection may not occur more frequently than two times per week.
**3-Noninvasive Collection of Biological Specimens:** Collected prospectively for research purposes by noninvasive means:<br>(1) Hair and nail clippings in a non-disfiguring manner.<br>(2) Deciduous teeth at time of exfoliation or if routine patient care indicates a need for extraction.<br>(3) Permanent teeth if routine patient care indicates a need for extraction.<br>(4) Excreta and external secretions (including sweat).<br>(5) Uncannulated saliva collected either in an unstimulated fashion or stimulated by chewing gumbase or wax or by applying a dilute citric solution to the tongue.<br>(6) Placenta removed at delivery.<br>(7) Amniotic fluid obtained at the time of rupture of the membrane prior to, or during, labor.<br>(8) Supra- and subgingival dental plaque and calculus, provided the collection procedure is not more invasive than routine prophylactic scaling of the teeth and the process is accomplished in accordance with accepted prophylactic techniques.<br>(9) Mucosal and skin cells collected by buccal scraping or swab, skin swab, or mouth washings.<br>(10) Sputum collected after saline mist nebulization.

EXPEDITE CATEGORIES
**4-Noninvasive Collection of Data:** Data must be collected through noninvasive procedures (not involving general anesthesia or sedation) routinely employed in clinical practice, excluding procedures involving x-rays or microwaves.<br>(1) Physical sensors that are applied either to the surface of the body or at a distance and do not involve input of significant amounts of energy into the subject or an invasion of the subject's privacy.<br>(2) Weighing the subject.<br>(3) Testing sensory acuity.<br>(4) Magnetic resonance imaging (MRI).<br>(5) Electrocardiography, electroencephalography, thermography, detection of naturally occurring radioactivity, electroretinography, ultrasound, diagnostic infrared imaging, Doppler blood flow, and echocardiography.<br>(6) Moderate exercise, muscular strength testing, body composition assessment, and flexibility testing, where appropriate, given the age, weight, and health of the individual.
**5-Collected Material:** Research involves:<br>(1) Materials (data, documents, records, or specimens) that have been collected for any purpose, including previous research; or<br>(2) Materials (data, documents, records, or specimens) that will be collected solely for nonresearch purposes (such as medical treatment or diagnosis).
**6-Collection of Data From Voice, Video, or Photographs:** Research involves collection of data from voice, video, or photographs.
**7-Group Characteristics, Surveys, Interviews, and Quality Assurance:** Research must be on individual or group characteristics or behavior (including, but not limited to: research on perception, cognition, motivation, identity, language, communication, cultural beliefs or practices, and social behavior), or will employ survey, interview, oral history, focus group, program evaluation, human factors evaluation, or quality assurance methodologies. ***NOTE****: Some research in this category may be exempt from the VA regulations for the protection of human subjects (38 CFR 16.101(b)(2) and (b)(3)). This listing refers only to research that is not exempt.*
