# Supplementary material for: Implementation of Group Physical Therapy for Knee Osteoarthritis: A Cluster Randomized Clinical Trial
Source: JAMA Netw Open. 2025 Oct 2;8(10):e2535038. doi: 10.1001/jamanetworkopen.2025.35038 (PMC12492051; doi:10.1001/jamanetworkopen.2025.35038)
Supplement: Supplement 2. — eTable 1. Description of Foundational and Enhanced Implementation Support Packages eTable 2. Estimated Means by Arm and Estimated Differences in Site-Level Implementation Outcomes During Months 1 to 12 Between Arms With Associated 95% CIs From Generalized Linear Models eFigure. Adoption Status and First Group PT Class Delivered by Month eTable 3. Estimated Mean at Last Class (n = 130 With Last Class) by Arm and Estimated Difference in Outcomes Between Enhanced Support vs Foundation Support With Associated 95% CIs From Generalized Linear Models eTable 4. Estimated Mean at Baseline and 7 Weeks for Patient Outcomes and Estimated Mean Difference in Change in Outcomes Between Arms With Associated 95% CIs When Including All Class Data up to 100 Days After First Class From Linear Mixed Models [file jamanetwopen-e2535038-s002.pdf]

Supplementary Online Content

Allen KD, Webb S, Coffman CJ, et al. Implementation of group physical therapy for knee osteoarthritis: a cluster randomized clinical trial. *JAMA Netw Open*. 2025;8(10):e2535038. doi:10.1001/jamanetworkopen.2025.35038

eTable 1. Description of Foundational and Enhanced Implementation Support Packages

eTable 2. Estimated Means by Arm and Estimated Differences in Site-Level Implementation Outcomes During Months 1 to 12 Between Arms With Associated 95% CIs From Generalized Linear Models

eFigure. Adoption Status and First Group PT Class Delivered by Month

eTable 3. Estimated Mean at Last Class (n = 130 With Last Class) by Arm and Estimated Difference in Outcomes Between Enhanced Support vs Foundation Support With Associated 95% CIs From Generalized Linear Models

eTable 4. Estimated Mean at Baseline and 7 Weeks for Patient Outcomes and Estimated Mean Difference in Change in Outcomes Between Arms With Associated 95% CIs When Including All Class Data up to 100 Days After First Class From Linear Mixed Models

This supplementary material has been provided by the authors to give readers additional information about their work.

© 2025 Allen KD et al. *JAMA Network Open*.

eTable 1. Description of Foundational and Enhanced Implementation Support Packages

Package	Activity	Description
**Foundational Support** (resources available to all sites for full study period)	Toolkit	Standardized program materials and training curriculum to educate delivery teams about the Group PT intervention and implementation process, including recorded webinars, implementation handbook, and Group PT delivery guide.
	SharePoint	Secure SharePoint for access to Group PT implementation support materials (e.g., patient materials, marketing templates, guides for documenting patient outcomes), and standardized materials to facilitate monitoring sites' progress.
	Pre-developed EHR templates	Consult, initial evaluation and class participation EHR templates developed with clinical guidance to facilitate Group PT delivery and collection of patient-reported outcomes.
	Data reports	Monthly reports to assist sites with tracking their implementation activity (e.g., patient enrollment, attendance, and satisfaction). Quarterly reports will be sent with patient outcomes (e.g., PROMIS scores and chair rise improvement).
	Learning Collaborative	Office hour calls and Microsoft Teams channel designed to capture and share local knowledge through networking. Office hour calls will be specific to each cohort of sites; they will include a short, structured presentation on a relevant topic to each phase of implementation plus unstructured time for sites to ask questions and give feedback to each other. An implementation specialist will facilitate office hours calls and contribute suggestions for addressing barriers or challenges expressed by sites. The Teams channel will provide an outlet to communicate asynchronously with individuals from different cohorts.
**Enhanced Support** (resources available to non-adopting and/or non- sustaining sites)	Foundational resources	No change to accessing resources outlined above as part of foundational support.
	Technical assistance	Direct access to Function QUERI implementation facilitators for technical assistance as needed.
	External facilitation	Tailored, one-on-one calls approximately every 3 to 4 weeks, with a maximum of 6 facilitation hours, between site implementation teams and a trained practice facilitator with the goal of promoting interactive problem solving in the context of a supportive interpersonal relationship. Discussion may focus on key barriers to implementation, available assets to leverage, and actionable tasks to monitor and improve delivery.

© 2025 Allen KD et al. *JAMA Network Open*.

eTable 2. Estimated means by arm and estimated differences in site-level implementation outcomes during months 1-12 between arms with associated 95% CI from generalized linear models

Outcome	Definition	Enhanced Support (n=10) Estimated Mean (95% CI)	Foundational Support (n=9) Estimated Mean (95% CI)	Enhanced vs. Foundational Support	
				Estimated Mean Difference (95% CI)	*p-value*
**Penetration**	*Average number of patients enrolled in Group PT monthly*	0.7 (0.2,1.1)	0.7 (0.1,1.2)	MD=-0.0 (-0.7, 0.7);	0.95
	Average number of patients enrolled	7.3 (4.0,13.5)	7.2 (3.4,15.1)	RR=1.0 (0.43, 2.42)	0.96
**Fidelity** [a]	*Average number of sessions attended by enrolled patients (6 classes max.)*	4.7 (4.0,5.4)	4.3 (3.5,5.1)	MD=0.4 (-0.5, 1.3)	0.33
	Average number of sessions attended by enrolled patients (all classes)	5.7 (4.0,7.4)	4.6 (2.6, 6.6)	MD=1.2 (-1.2, 3.5)	0.31

[a] Fidelity calculations only included sites that held a Group PT class (enhanced support sites=9 and foundational support sites=7)
MD= Mean Difference; RR= Rate Ratio
Secondary outcome analyses included 189 patients enrolled in Group PT during the full 12-month study period.

© 2025 Allen KD et al. *JAMA Network Open*.

eFigure. Adoption status and first Group PT class delivered by month

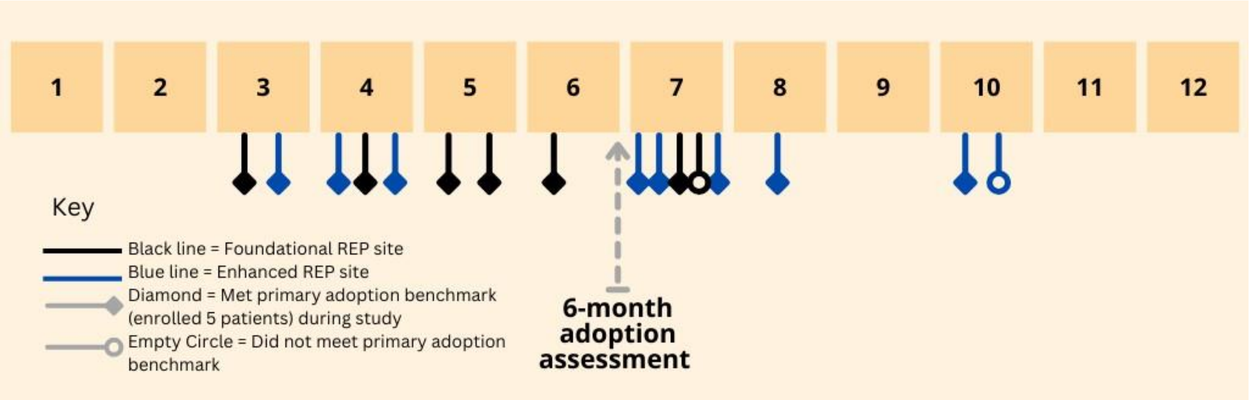

Figure includes 16 sites that launched a Group PT program. There were three sites that did not launch a Group PT program and are excluded from the figure.

© 2025 Allen KD et al. *JAMA Network Open*.

eTable 3. Estimated mean at last class (n=130 with last class) by arm and estimated difference in outcomes between Enhanced support vs. Foundation support with associated 95% CIs from generalized linear models

**Outcomes[a]**	**Enhanced Support** Estimated Mean (95% CI)	**Foundational Support** Estimated Mean (95% CI)	**Enhanced vs. Foundational**	
			Estimated Mean Difference in Change (95% CI)	*p-value*
**Patient Satisfaction (0-10)**	8.8 (8.2,9.3)	8.7 (8.3,9.3)	MD=0.1 (-0.5, 0.6)	0.79
**Ability to deal with daily knee problems compared to before starting Group PT, % Better**	53.8% (32.4%, 74.0%)	67.3% (44.2%, 84.3%)	OR=1.8 (0.6, 5.2)	0.30

[a]Missing n=22 Satisfaction Score (14 Enhanced support; 8 Foundational Support)
n=19 missing Knee Pain assessment (11 Enhanced Support; 8 Foundational Support)
MD=Mean Difference; OR=Odds Ratio; CI=Confidence Interval

© 2025 Allen KD et al. *JAMA Network Open*.

eTable 4. Estimated mean baseline and 7 weeks for patient outcomes and estimated mean difference in change in outcomes between arms with associated 95% CIs when including all class data up to 100 days after first class from linear mixed models

Outcomes	Enhanced Support (n=66)[a]			Foundational Support(n=76)[a]			Enhanced vs. Foundational Support	
	Baseline Mean	Last Class Mean	Change (Last class-First Class (Mean, 95% CI)	Baseline Mean	Last Class Mean	Change (Last class-First Class (Mean, 95% CI)	Estimated Mean Difference in Change (95% CI)	p-value
PROMIS Pain Interference	61.1	58.3	-2.8 (-3.7,-1.9)	59.7	57.0	-2.7 (-3.9,-1.6)	-0.1 (-1.7,1.5)	0.91
PROMIS Physical Function	37.7	40.2	2.3 (1.3,3.3)	38.7	40.4	1.7 (0.5,2.9)	0.8 (-0.9,2.4)	0.37
Chair Rise Repetitions	10.1	12.6	2.6 (1.9,3.4)	10.1	13.0	3.0 (2.0,3.0)	-0.4 (-1.7,0.9)	0.55
Max Pain During Chair Rise	4.5	4.1	-0.4 (-0.8,1.0)	3.6	3.5	-0.2 (-0.7,0.4)	-0.2 (-0.9,0.5)	0.53

[a]N=16 sites (n=9 Enhanced Support; n=7 Foundational support) with patient participants; 2 patients in enhanced support did not have first class data available

© 2025 Allen KD et al. *JAMA Network Open*.
