# Supplementary material for: Implementation of Group Physical Therapy for Knee Osteoarthritis: A Cluster Randomized Clinical Trial
Source: JAMA Netw Open. 2025 Oct 2;8(10):e2535038. doi: 10.1001/jamanetworkopen.2025.35038 (PMC12492051; doi:10.1001/jamanetworkopen.2025.35038)
Supplement: Supplement 3. — Data Sharing Statement [file jamanetwopen-e2535038-s003.pdf]

Data Sharing Statement

Allen. Implementation of Group Physical Therapy for Knee Osteoarthritis. *JAMA Netw Open*. Published October 02, 2025. doi:10.1001/jamanetworkopen.2025.35038

Data
Additional Information: NCT05282927
Data available: Yes
Data types: Deidentified participant data
How to access data: Kelli.Allen@va.gov
When available: With publication

Supporting Documents
Document types: None

Additional Information
Who can access the data: De-identified data will be made available as allowable under the regulations and policies of study sponsors.
Types of analyses: For abstracts, manuscripts or other dissemination products
Mechanisms of data availability: After approval of a proposal
